# S6K1 is a Targetable Vulnerability in Tumors Exhibiting Plasticity and Therapy Resistance

**DOI:** 10.7150/ijbs.96672

**Published:** 2025-01-01

**Authors:** Saptadwipa Ganguly, Ravshan Burikhanov, Vitaliy M. Sviripa, Sally Ellingson, Jieyun Jiang, Christian M. Gosser, David Orren, Eva M. Goellner, Gautham G. Shenoy, Mahadev Rao, John D'Orazio, Christine F. Brainson, Chang-Guo Zhan, Peter H. Spielmann, David S. Watt, Vivek M. Rangnekar

**Affiliations:** 1 Department of Toxicology and Cancer Biology, College of Medicine, University of Kentucky, Lexington, Kentucky, USA.; 2 Department of Radiation Medicine, College of Medicine, University of Kentucky, Lexington, Kentucky, USA.; 3 Department of Molecular and Cellular Biochemistry and Molecular Biology, College of Medicine, University of Kentucky, Lexington, Kentucky, USA.; 4 Division of Internal Medicine, College of Medicine, University of Kentucky, Lexington, Kentucky, USA.; 5 Department of Pediatrics, College of Medicine, University of Kentucky, Lexington, Kentucky, USA.; 6 Markey Cancer Center, University of Kentucky, Lexington, Kentucky, USA.; 7 Department of Pharmaceutical Sciences, College of Pharmacy, University of Kentucky, Lexington, Kentucky, USA.; 8 Molecular Modeling and Pharmaceutical Center, College of Pharmacy, University of Kentucky, Lexington, Kentucky, USA.; 9 Department of Pharmaceutical Chemistry, Manipal College of Pharmaceutical Sciences, Manipal Academy of Higher Education, Manipal, India.; 10 Department of Pharmacy Practice, Center for Translational Research, Manipal College of Pharmaceutical Sciences, Manipal Academy of Higher Education, Manipal, India.

**Keywords:** apoptosis, RPS6KB1/S6K1, ebastine, plasticity, therapy resistance

## Abstract

**Background:** Most tumors initially respond to treatment, yet refractory clones subsequently develop owing to resistance mechanisms associated with cancer cell plasticity and heterogeneity.

**Methods:** We used a chemical biology approach to identify protein targets in cancer cells exhibiting diverse driver mutations and representing models of tumor lineage plasticity and therapy resistance. An unbiased screen of a drug library was performed against cancer cells followed by synthesis of chemical analogs of the most effective drug. The cancer subtype target range of the leading drug was determined by PRISM analysis of over 900 cancer cell lines at the Broad Institute, MA. RNA-sequencing and enrichment analysis of differentially expressed genes, as well as computational molecular modeling and pull-down with biotinylated small molecules were used to identify and validate RPS6KB1 (p70S6K or S6K1) as an essential target. Genetic restoration was used to test the functional role of S6K1 in cell culture and xenograft models.

**Results:** We identified a novel derivative of the antihistamine drug ebastine, designated Super-ebastine (Super-EBS), that inhibited the viability of cancer cells representing diverse KRAS and EGFR driver mutations and models of plasticity and treatment resistance. Interestingly, PRISM analysis indicated that over 95% of the diverse cancer cell lines tested were sensitive to Super-EBS and the predicted target was the serine/threonine kinase S6K1. S6K1 is upregulated in various cancers relative to counterpart normal/benign tissues and phosphorylated-S6K1 predicts poor prognosis for cancer patients. We noted that inhibition of S6K1 phosphorylation was necessary for tumor cell growth inhibition, and restoration of phospho-S6K1 rendered tumor cells resistant to Super-EBS. Inhibition of S6K1 phosphorylation by Super-EBS induced caspase-2 dependent apoptosis *via* inhibition of the Cdc42/Rac-1/p-PAK1 pathway that led to actin depolymerization and caspase-2 activation. The essential role of S6K1 in the action of Super-EBS was recapitulated in xenografts, and knockout of S6K1 abrogated tumor growth in mice.

**Conclusion:** S6K1 is a therapeutic vulnerability in tumors exhibiting intrinsic and/or acquired resistance to treatment

## Introduction

Resistance to standard-of-care treatments is a leading cause of cancer-related deaths 1. Although some tumors may exhibit pre-existing or intrinsic resistance, most tumors initially respond to treatments but subsequently develop acquired resistance. The mechanisms underpinning tumor resistance are complex and include genetic and/or epigenetic changes which may not always be druggable, including increased drug efflux, drug target alterations, drug inactivation, DNA damage repair, and cell-death inhibition 1, 2. Importantly, increased growth kinetics contribute to increased genomic instability, tumor cell heterogeneity and epithelial-mesenchymal transition and lead to emergence of treatment resistant tumor clones 3, 4. Additionally, tumor cells may undergo neuroendocrine differentiation (NED) and acquire neural and endocrine features often associated with rapidly growing and highly aggressive phenotypes 5-7. These features are also associated with resistance to conventional treatments and are especially noted in lung, prostate and breast cancers 5-7. There are also reports of intrinsic and acquired resistance to immunotherapy through mechanisms such as aberrant expression of tumor antigens, changes in immunogenicity, alterations in the antigen presentation pathways and factors present in the local microenvironment 8. As a result, neither precision chemotherapy nor immunotherapy, which is dependent on the tumor mutation burden associated with tumor plasticity and heterogeneity, can consistently provide durable responses in many cases. Accordingly, significant improvements are needed in the development of drugs that target therapy resistant tumors.

Lung cancer, for example, is a leading cause of cancer mortality in the world 9. Mutations in the Kirsten rat sarcoma virus (KRAS) oncoprotein and epidermal growth factor receptor (EGFR) are found in approximately 60% of non-small cell lung cancer (NSCLC) patients 10, 11. Mutant KRAS protein is among the most common oncogenes associated with a poor prognosis for patients with NSCLC. Mutant KRAS, is a challenging target for multiple reasons, including the structural variations that lack stable binding sites and its affinity for guanosine triphosphate/guanosine diphosphate (GTP/GDP) 12. Although a recent breakthrough in the identification of drugs such, as sotorasib and adagrasib for advanced metastatic lung cancer patients with KRAS G12C mutation engendered optimism for targeting mutant KRAS, tumors with intrinsic or acquired resistance to the G12C inhibitors are also reported 13. Similarly, a large number of lung tumors have activating mutations in the EGFR protein that renders them oncogenic and resistant to tyrosine kinase inhibitors 14. Osimertinib was developed to target mutations in EGFR including T790M by forming a covalent bond to the C797 residue in the ATP-binding site and thereby preventing its activation. However, resistance to osimertinib quickly develops following treatment through multiple mechanisms in lung cancer patients 15, 16.

As another example, prostate cancer is the second leading cause of cancer related deaths in men, and patients who fail Androgen Deprivation Therapy (ADT) often respond well to androgen receptor (AR) signaling inhibitors such as enzalutamide and abiraterone 17. Binding of enzalutamide to AR in the cytoplasm inhibits AR translocation into the nucleus, and in the nucleus, enzalutamide inhibits AR binding to the DNA and thereby blocks the transcription of genes associated with tumor progression and survival 18. However, although these patients initially respond to the AR signaling inhibitors, tumors quickly develop resistance to these drugs 15, 19-22. In summary, despite the initial success with new treatments for cancer resistance, tumor cells often become refractory to these therapies.

Tumor cell plasticity and heterogeneity rank high among the causes underlying treatment resistance 4. Intra-tumoral heterogeneity involves the existence of subpopulations of cancer cells that exhibit distinct genotypic and phenotypic alterations that contribute to poor clinical outcome. This scenario is further aggravated by epigenetic changes in the tumor microenvironment, especially the cancer-associated stromal cell population that includes heterogeneous group of infiltrating immune cells especially T-cells and myeloid-derived suppressor cell population 23. Tumor plasticity that enables cells to acquire new phenotypic and functional features is an inherent trait that allows transitioning of the tumor cells among distinct cell states. Tumor plasticity, which is one of the mechanisms leading to tumor cell heterogeneity, contributes to the critical alterations associated with tumor initiation, progression and metastasis, as well as therapeutic resistance. The most prominent and consequential examples of tumor cell plasticity are epithelial-mesenchymal transition (EMT), mesenchymal-epithelial transition (MET) and NED of tumor cells 24. EMT and its reversal to MET are critical in initiating tumor cell migration from the primary site and establishing metastasis at distant sites 25. NED of tumor cells features architectural and cytologic alterations reminiscent of non-neoplastic neuroendocrine cells and is often noted in lung, prostate and breast cancer cell 5-7, 24. Metastasis and NED are usually associated with resistance to standard-of-care treatment and high mortality rates in patients 26-28. Some of the underlying molecular mechanisms associated with treatment resistance include the induction of drug translocators that prevent the accumulation of optimal drug concentrations at the target sites in tumors 1, as well as compensatory mutations in the target protein and induction of anti-cell death proteins, effecting the anti-apoptotic/cell survival pathways 29.

As part of an effort to identify drugs that may target therapy-resistance in tumor cells, we screened an FDA-approved drug library of approximately 1400 compounds. This screen led to the identification of an antihistamine drug ebastine (EBS) 30 with the desired function. Our efforts led to the synthesis and evaluation of a potent analog of ebastine, we designated as Super-ebastine (Super-EBS) that inhibits the growth of diverse heterogeneous cancer cell lines and models of tumor cell plasticity and drug resistance. Subsequent efforts identified that Super-EBS targets Ribosomal S6 kinase 1, RPS6KB1 (S6K1), a key downstream kinase in the mammalian target of rapamycin complex 1 (mTORC1) pathway that regulates ribosome biogenesis and protein translation 31. S6K1 expression is upregulated in lung adenocarcinoma, lung squamous cell carcinoma, prostate cancer, hepatocellular carcinoma, colon cancer, esophageal cancer and head and neck squamous cell carcinoma 32. In addition, patients treated with radiotherapy who had low phospho-S6K1 (p-S6K1) expression in their breast tumors had significantly higher loco-regional recurrence-free survival than patients with high p-S6K1 expression. Increased p-S6K1 may be associated with radio-resistance in breast cancer stem cells 33 and high p-S6K1 expression is associated with poor 5-year survival in NSCLC 34. S6K1 is reported to be associated with therapy resistance in ER- positive breast and cervical cancer to palbociclib and cisplatin, respectively 35, 36. Resistance to tyrosine kinase inhibitors of EGFR in NSCLC is driven by constitutive activation of S6K1, with p-S6K1 levels higher in the gefitinib-resistant lung cancer cells and PDX tumors compared to the gefitinib-sensitive lung cancer and parental PDX tumors, respectively. Knock-down of S6K1 leads to sensitization of the gefitinib-resistant cells to gefitinib 37. The mTOR inhibitors rapamycin and rapalogs indirectly inhibit p-S6K1, which is a downstream substrate of the mTORC1 complex containing mTOR. However, these mTOR inhibitors also activate Akt through an IGF-1R-dependent feedback mechanism that reactivates the mTORC1/p-S6K1 pathway, ultimately conferring resistance to these drugs 38. Also, mTORC1 inhibition leads to activation of autophagy that promotes the survival of cancer cells. Given the obvious advantages of directly inhibiting S6K1 phosphorylation, we examined p-S6K1 as a target in tumors exhibiting intrinsic or acquired resistance and in models portraying tumor plasticity- neuroendocrine differentiation and heterogeneity exemplified in PRISM analysis of diverse cancer cell lines. We report the identification of p-S6K1 as a target of Super-EBS that leads to growth inhibition of tumor cells through activation of caspase-2 dependent apoptotic cell death.

## Materials and Methods

### Cell lines

Human prostate cancer cells (LNCaP, PC-3 and DU145), lung cancer cells (A549 and NCI-H1299), human lung fibroblast cells (HEL), and human breast cancer cells (MCF-7 and MDA-MB-231) were obtained from ATCC (Gaithersburg, MD). NCI-H1975 (H1975) human lung cancer cells were generously provided by Carla F. Kim (Harvard Medical School, Boston, MA); NCI-H1650 (H1650), NCI-H2009, PC-9 and osimertinib-resistant PC-9-OR developed by increasing dosage of osimertinib starting from 0.007 μM over time, were generously provided by Christine F. Brainson (University of Kentucky, Lexington, KY) and validated by STR genotyping. Taxane-resistant A549TR cells were generously provided by Bruce Zetter (Memorial Sloan Kettering Cancer Center, Manhattan, NY). Mouse KP7B lung adenocarcinoma cells that maintain the disease relevance of lung cancer models, were derived from mutant KRAS and p53-deleted lung tumors. These were induced by the Cre-adenovirus in C57BL/6 KRAS^G12D/+^; p53^-/-^ mice, were generously provided by Professor Tyler Jacks' laboratory (Massachusetts Institute of Technology, Cambridge, MA) 39. Human prostate cancer cells C4-2 and enzalutamide resistant-C4-2R were generously provided by Xiaoqi Liu (University of Kentucky, Lexington, KY). LNCaP derivative enzalutamide-resistant cells (M49F) were generously provided by Dr. Amina Zoubeidi (University of British Columbia, Vancouver, BC, Canada). CwR22Rv1 cells were generously provided by Dr. Tom Pretlow (Case Western Reserve School of Medicine, Cleveland, OH).

### Computational modeling and methods

We used AlphaFold predicted structure of the complete protein sequence provided through the AlphaFold protein structure database and an experimental structure (PDB code 4L46) in our modeling. PDB 4L46 was chosen as it was made to study the hydrophobic motif which contains Thr389. Binding sites were predicted using MOE's Site Finder tool. The AlphaFold structure had four binding sites with a good PLB score (propensity for ligand binding). The experimental structure only had one binding site with a significant PLB score which overlays where the PF-4708671 inhibitor is bound. In each model, the binding of PF-4708671 and Super-EBS were both tested and Super-EBS was predicted as a better binder across all models.

Binding in the AlphaFold structure with the 4^th^ predicted binding site was of interest due to the presence of Thr389 in the binding site. For comparison, EBS was also docked in this same binding site.

### Neuroendocrine differentiation

A549 and LNCaP cells were seeded in 6-well plates, one set as a control and the other for differentiation. After 24 h, the cells were washed with 1X PBS twice. IBMX (3-isobutyl-1-methylxanthine) (I5879, Sigma) and forskolin (1099, R&D Systems) at 0.5 mM in non-FBS containing media were used to induce neuroendocrine differentiation in A549 cells. Cells in non-FBS containing media were used as controls. After 96 h, the cells were harvested and tested for markers of neuroendocrine differentiation (NED)-neuron-specific enolase (NSE) or βIII Tubulin. LNCaP cells were transdifferentiated using 50 ng/mL of IL-6 (570806, BioLegend) in charcoal-stripped serum containing medium for 6 days. Cells with only charcoal-stripped serum containing medium were used as the corresponding control. After 6 days, the cells were collected for testing for NED markers. The cells were imaged at the end of the differentiation period using EVOS microscope (Life Technologies, AMEP-VH009). The experiments were performed in triplicate and neurite outgrowth was quantified with the help of ImageJ's software, with the NeuronJ plugin, tracing the neurites present in 20 cells from each image.

### Supplemental methods

Information regarding chemicals, plasmids and list of antibodies; cell viability and apoptosis assays; kinase antibody array assay; transfection; generation of CRISPR/Cas knockout cells; PRISM analysis; fluorescence microscopy; RNA sequencing and enrichment analysis of differentially expressed genes; real-time quantitative PCR analyses; and animal studies is provided in Supplementary Materials.

### Statistical analysis

All experiments were performed in triplicates to verify reproducibility of the observations and the data are presented as the mean of at least 3 experiments ± standard deviations (SD). The graphs were generated using GraphPad Prism. The *p* values were calculated using Student's *t*-test.

## Results

### Super-EBS inhibits the growth of diverse cancer cells

**Figure 1:**
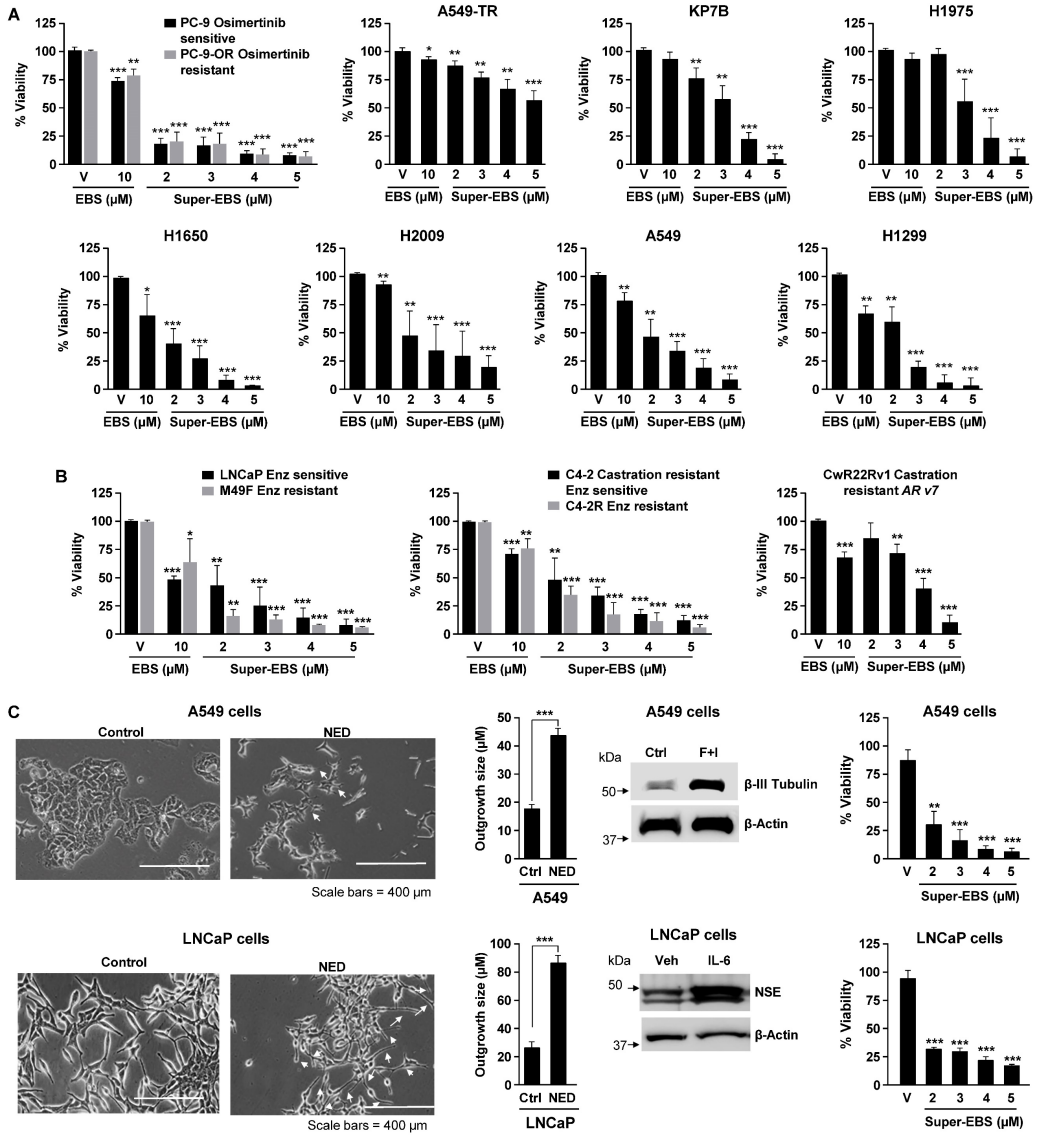
** Super-EBS is more potent than EBS in inhibition of tumor cell growth. (A)** Super-EBS is more effective than EBS in growth inhibition of drug-sensitive and -resistant lung cancer cells. Various lung cancer cells were treated with the indicated concentrations of Super-EBS or EBS (10 µM) for 24 h and cell viability was quantified by resazurin assays. PC9-OR cells with acquired resistance to osimertinib were maintained in the presence of 0.5 µM osimertinib, and A549TR cells resistant to taxanes and were maintained in 0.5 µM paclitaxel and tested for growth inhibition by Super-EBS in the absence of osimertinib or paclitaxel, respectively. **(B)** Super-EBS is more effective than EBS in growth inhibition of drug-sensitive and -resistant prostate cancer cells. Prostate cancer cells LNCaP and their enzalutamide-resistant derivative cells M49F (resistant to 10 µM enzalutamide), castration-resistant prostate cancer cells C4-2 and their Enzalutamide-resistant derivative cells C4-2R (resistant to 20 µM enzalutamide) and CwR22Rv1 (resistant to abiraterone and enzalutamide) were treated with Super-EBS or EBS for 24 h and cell viability determined by resazurin assays. **(C)** Super-EBS induces growth inhibition of cancer cells that have undergone neuroendocrine differentiation (NED). A549 or LNCaP cells were induced to undergo NED by treatment with forskolin + IBMX or IL-6. Light microscopy images of the cells are shown (Left Panels). Scale bar, 400 µm. The length of the neurite-outgrowth was measured using NeuronJ plugin after tracing the neurites from 20 cells in each image (Middle Panels). Whole-cell lysates were then examined by western blot analysis for NED marker β-III tubulin or neuron-specific enolase (NSE) (Middle Panels). The differentiated cells were treated with different concentrations of Super-EBS for 24 h and cell viability was determined by resazurin assays (Right Panels). (A-C) Mean ± SD from three independent experiments are shown. *P ≤ 0.05, **P ≤ 0.01, ***P ≤ 0.001 by the Student's *t*-test.

To identify small molecules that inhibited the growth of cancer cells with intrinsic or acquired resistance to therapy, we screened a library of FDA-approved compounds. This screen identified ten drugs (**Table S1**; **Fig. S1A**) that induced growth inhibition when used at 10 μM concentrations in A549TR or PC-9-OR cells lung cells that have acquired resistant to paclitaxel or osimertinib (**Fig. S1B**), respectively, as well as KP7B cells that express mutant KRAS and lack p53, rendering them intrinsically resistant to DNA damaging chemotherapeutic agents 40. As seen in **Fig. S1A**, the antihistamine drug ebastine (EBS) was most effective in inhibiting the growth of these three lung cancer cell lines. Although ebastine was previously shown to inhibit the growth of lung cancer cells at an IC_50_ exceeding 15 μM, its effects on diverse therapy resistant cancer cells have not been reported. In view of the potentially high IC_50_ of ebastine, we undertook the chemical modification of ebastine to generate analogs that are both superior in potency than ebastine and extend the sensitivity range of the cancer cell types. Before we proceeded with the generation of analogs it was important to determine if other antihistamines that belong to the same family as ebastine had similar or better potency in killing cancer cells. As seen in **Fig. S1C**, ebastine (10 µM) was the most effective antihistamine drug in killing cancer cells. We next synthesized and tested various analogs of ebastine described in **Fig. S1D, E.** Interestingly, an ebastine analog that contains an aminoguanidine sidechain (T41614) was most effective in inhibiting the growth of cancer cell lines. We designated this analog as Super-EBS (Super-EBS). As seen in **Fig. 1A**, lung cancer cells with driver mutations in KRAS (A549) and EGFR (H1975, H1650, PC-9), as well as cells with intrinsic resistance to chemotherapeutic agents due to mutant RAS and p53 loss (KP7B, H2009 and H1299) or acquired resistance to chemotherapy (PC9-OR and A549TR) were more sensitive to Super-EBS than to EBS. To determine whether the effect of Super-EBS was limited to lung cancer cells, we tested prostate cancer cells that were androgen-responsive (LNCaP) or castration resistant prostate cancer cells derived from LNCaP cells (C4-2), as well as their enzalutamide-resistant derivatives (M49F and C4-2R) for their relative sensitivity to Super-EBS versus EBS. As seen in **Fig. 1B**, Super-EBS was more potent in inducing growth inhibition in all the prostate cancer cell lines relative to EBS. Moreover, Super-EBS showed growth inhibitory effects in prostate cancer cells CWR22Rv1 (**Fig. 1B**) that are intrinsically resistant to abiraterone and enzalutamide 41. We also tested whether Super-EBS induces growth inhibition of cancer cells that have undergone neuroendocrine differentiation (NED). As seen in **Fig. 1C**, A549 or LNCaP cells that were induced to undergo NED showed dose-dependent inhibition with Super-EBS. Moreover, androgen receptor-negative prostate cancer cells PC-3 and DU145, estrogen-receptor positive breast cancer cells MCF7 and triple-negative breast cancer cells MDA MB 231 were also sensitive to Super-EBS (**Fig. S1F**). Super-EBS causes only marginal loss of viability in normal lung epithelial cells (Beas2B) and normal prostate epithelial cells (RWPE-1) at the highest drug concentration (**Fig. S1G**). Super-EBS was more effective than ebastine in inhibiting therapy-resistant cancer cells. Additionally, the aminoguanidine sidechain of Super-EBS or carebastine, that binds to the H1 receptor for histamine, did not show growth inhibitory activity (**Fig. S1H**).

We also performed high-throughput analysis of the effect of Super-EBS on viability of over 900 cell lines from over 45 cancer subtypes using PRISM analysis (Broad Institute, MA). As seen in the heatmap (**Fig. S1I**), Super-EBS caused 80% or more growth inhibition in over 95% of the cancer cell lines in the panel at a concentration of 3.3 μM, implying activity against a wide range of cancer cells that are genotypically and phenotypically heterogeneous. Collectively, these findings indicate that Super-EBS is a superior analog of ebastine in inhibiting the growth of a heterogeneous panel of cancer cells, including those that have intrinsic or acquired resistance to therapeutic compounds.

### Super-EBS induces cancer cell death via the caspase-2 apoptotic pathway

**Figure 2:**
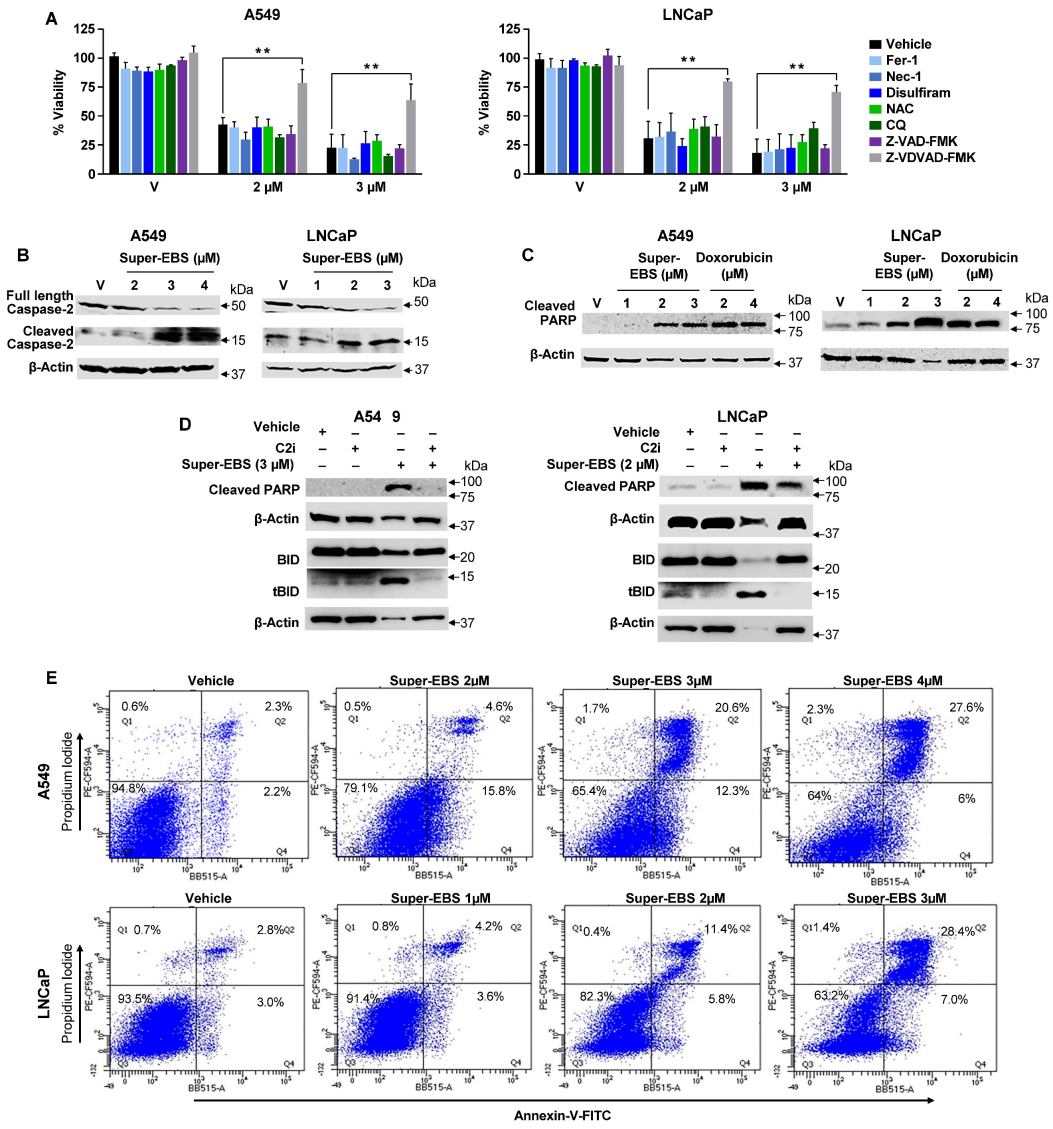
** Super-EBS mediates caspase-2 dependent apoptotic cell death. (A)** Caspase-2 inhibitor prevents Super-EBS mediated cell death. Lung cancer A549 and prostate cancer cells LNCaP were pre-treated for 1 h with vehicle (V) or specific inhibitors of the lysosome-based cell death pathways including ferroptosis (Fer-1, 2 µM), necroptosis (Nec-1, 10 µM), reactive oxygen species generation (NAC, 1 mM), pyroptosis (Disulfiram, 10 µM), and autophagy (Chloroquine, 25 µM), and apoptosis pathways excluding caspase-2 (zVAD-fmk,10 µM) and specific for caspase-2 (Z-VDVAD-FMK, 20 µM). The cells were then treated with different concentrations of Super-EBS for 24 h. Cell viability was then quantified by resazurin assays. Mean ± SD from three independent experiments is shown. **P ≤ 0.01 by the Student's *t*-test. **(B)** Super-EBS causes dose dependent activation of caspase 2. A549 and LNCaP cells were treated with vehicle (V) or the indicated concentrations of Super-EBS for 12 h, and whole-cell lysates were analyzed by western blot for full length and cleaved capsase-2. **(C)** Super-EBS causes cleavage of caspase-2 substrate PARP. A549 and LNCaP cells were treated with vehicle (V) or the indicated concentrations of Super-EBS or doxorubicin for 24 h. Whole cell lysates were probed for cleaved PARP by western blot. **(D)** Caspase-2 inhibitor prevents PARP and BID cleavage induced by Super-EBS. A549 and LNCaP cells were treated with vehicle (V) or Super-EBS in the presence or absence of caspase-2 inhibitor (C2i) (20 µM) for 24 h. After 24 h, whole-cell lysates were subjected to western blot analysis for caspase-2 substrates, PARP and BID. **(E)** Quantitation of apoptosis by Super-EBS. A549 and LNCaP cells were treated with vehicle or Super-EBS at the indicated concentrations for 24 h and the cells were subjected to Annexin V/Propidium iodide (PI) staining and flow cytometry to quantify early and late apoptosis cells in the Flow Cytometry and Immune Monitoring Shared Resource Facility.

To determine if the Super-EBS mediated loss of viability occurred through the histamine H1 receptor, we tested the action of Super-EBS in the presence of control and two different H1 receptor antibodies (from two different sources) as seen in **Fig. S2A**. The H1 receptor antibodies did not inhibit the action of Super-EBS suggesting that Super-EBS does not carry out its action through the H1 receptor (**Fig. S2A**). Next, to identify the mechanism by which Super-EBS inhibits the growth of cancer cells, we pre-treated lung and prostate cancer cells with pharmacological inhibitors of various cell death pathways and then tested the cell cultures for growth inhibition by Super-EBS using resazurin assays. The specific inhibitors of the lysosome-based cell death pathways such as ferroptosis, necroptosis, reactive oxygen species generation, pyroptosis, and autophagy, as well as caspase-2 dependent or caspase-3 dependent apoptosis is depicted in **Fig. S2B**. As seen in **Fig. 2A**, growth inhibition of the cancer cells by Super-EBS was prevented by the caspase-2 inhibitor but not by any other cell death pathway inhibitor. These findings suggested that the caspase-2 cell death pathway may be involved in the growth inhibitory action of Super-EBS.

The formation of PIDDosome consisting of the proteins PIDD, RAIDD and caspase-2, in response to certain apoptotic insults leads to dimerization of caspase-2 followed by its cleavage to produce activated caspase-2 42. To confirm that Super-EBS activates caspase-2, we treated A549 and LNCaP cells with vehicle or Super-EBS and we performed western blot analysis for full length and cleaved capsase-2. As seen in **Fig. 2B**, Super-EBS induced dose-dependent decrease in full-length caspase-2 and an increase in cleaved caspase-2, indicative of the activation of caspase-2.

As Poly (ADP-ribose) polymerases (PARP) and BH3 interacting domain death agonist (BID) serve as substrates of activated caspase-2 43, we tested whether Super-EBS induces PARP and BID cleavage, and whether this action of Super-EBS was blocked by caspase-2 inhibition. A549 and LNCaP cells were treated with vehicle or Super-EBS and whole cell lysates were probed for cleaved PARP or tBID, the cleaved product of BID, by western blot analysis. Cells treated with doxorubicin were used as a control for PARP cleavage. As seen in **Fig. 2C**, Super-EBS or doxorubicin induced dose-dependent increase in cleaved PARP, indicative of apoptosis in response to the drugs relative to vehicle. Super-EBS also induced BID cleavage in A549 and LNCaP cells (**Fig. 2D; S2D**). Importantly, PARP and BID conversion to cleaved PARP and tBID, respectively, induced by Super-EBS was prevented by the caspase-2 inhibitor (**Fig. 2D**). These findings implied that Super-EBS induced the cleavage of PARP and BID by caspase-2 activation-dependent pathway.

Activation of caspase-2 leads to apoptosis by caspase-3 dependent as well as independent pathways 43, 44. Consistent with these observations, LNCaP and A549 cells treated with Super-EBS showed activation of caspase 3, and an increase in the number of apoptotic cells as judged by Annexin V/propidium iodide (PI) staining (**Fig. 2E**). These studies indicated that both caspase-2 and caspase-3 were activated by Super-EBS. However, as cell death induced by Super-EBS was prevented by the caspase-2 inhibitor but not caspase-3 inhibitor, our findings suggested that caspase-3 activation was dispensable, and caspase 2 activation was essential in the growth inhibitory action of Super-EBS.

### Super-EBS inhibits the activation of the cell survival kinase RPS6KB1 (S6K1)

**Figure 3:**
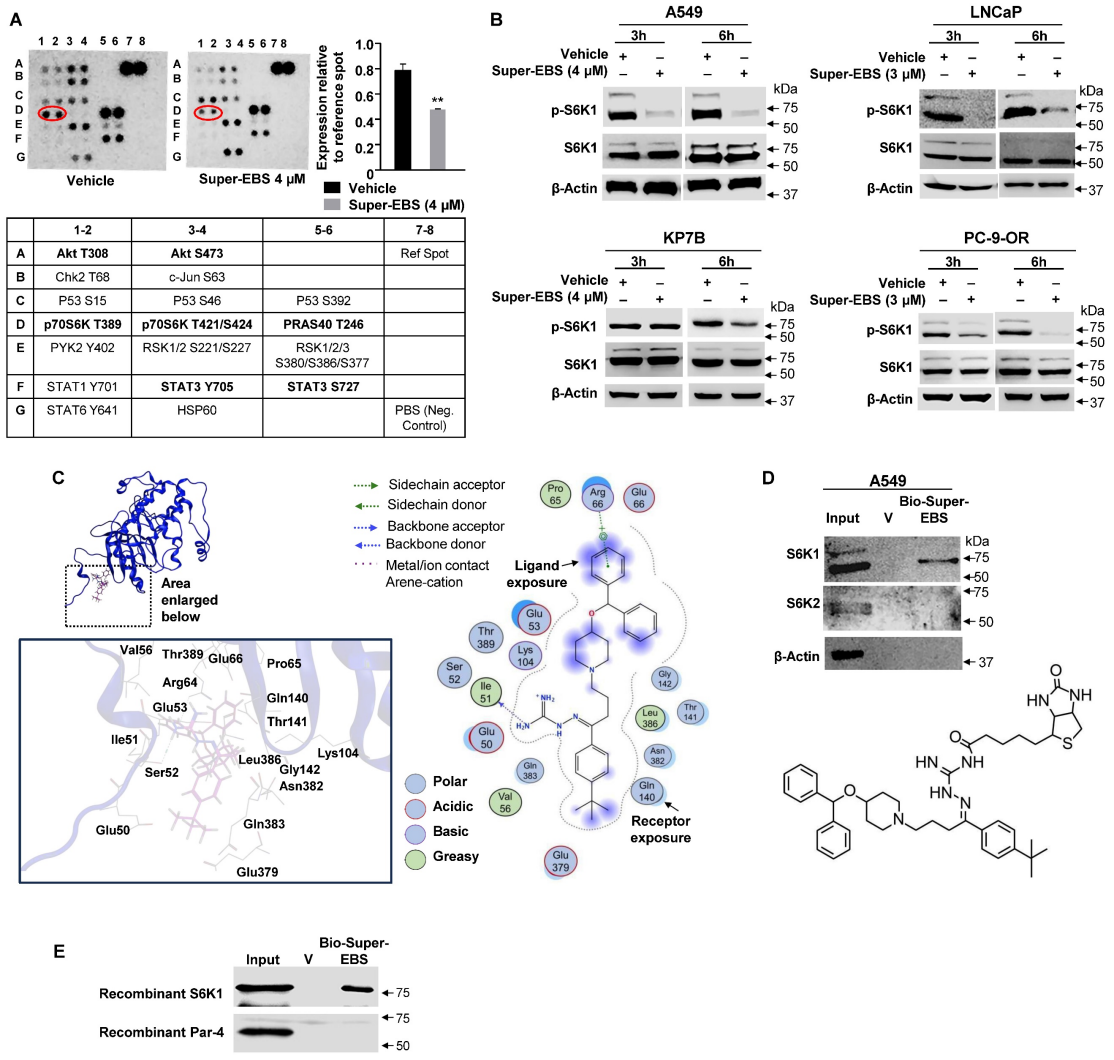
** Super-EBS inhibits the activation of the cell survival kinase RPS6KB1 (S6K1). (A)** Phospho-protein antibody array identified S6K1 as a potential target of Super-EBS. A549 cells were treated with vehicle or Super-EBS (4 µM for 6 h) and cell lysates were used to probe an array of phospho-protein antibodies as indicated in Methods. The spots D1, D2 which correspond to T389 site on p-S6K1 show a decrease in intensity with Super-EBS. Quantitative evaluation of spot intensity showed a significant decrease in the intensity of the p-S6K1 signal with Super-EBS treatment relative to that with vehicle treatment. Mean ± SD from three independent experiments is shown. **P ≤ 0.01 by the Student's *t*-test.** (B)** Validation of p-S6K1 inhibition by Super-EBS. Various cancer cell lines were treated with vehicle or Super-EBS for 6 h and whole-cell lysates were subjected to western blot analysis for p-S6K1 and total protein S6K1. Note inhibition of phosphor-S6K1 (p-S6K1) but not total S6K1 protein levels by Super-EBS in both the cell lines.** (C)** Super-EBS is predicted to bind to S6K1 by computational modeling. Super-EBS binding (dashed circle) to S6K1. Super-EBS binds near the T389 phosphorylation site on S6K1 and this interaction is expected to prevent S6K1 phosphorylation and activation. Key interactions among Super-EBS and S6K1 residues are shown.** (D)** Super-EBS binds to S6K1 but not S6K2. We synthesized biotinylated Super-EBS by linking the biotin moiety to the amino-guanidine side chain of Super-EBS. The chemical structure of Biotinylated Super-EBS is shown in the Lower Panel. Whole cell lysates of A549 cells were then incubated with vehicle, biotinylated-Super-EBS (Bio-Super-EBS) and streptavidin beads. Bound proteins were eluted from beads with D-biotin. Eluates were subjected to western blot analysis with S6K1 and S6K2 antibodies. Note Bio-Super-EBS pulls down S6K1 but not S6K2 (Upper Panel). **(E)** Super-EBS binds to recombinant S6K1 protein but not recombinant Par-4 protein. Recombinant S6K1 protein or Par-4 protein (4 µg) were incubated with vehicle, biotinylated-Super-EBS (Bio-Super-EBS) and streptavidin beads. Bound proteins were eluted from beads with D-biotin. Eluates were subjected to western blot analysis with S6K1 or Par-4 antibody.

To identify the gene expression changes associated with growth inhibition caused by Super-EBS compared to EBS, we performed RNA-Seq on A549 cells treated with Super-EBS (2 µM for 24 h) and ebastine (10 µM for 24 h). The profile of the differentially expressed genes (DEGs) of Super-EBS versus EBS is shown in **Fig. S3A**. GO and Reactome pathway enrichment analysis of the DEGs showed genes associated with regulation of protein translation were significantly downregulated with Super-EBS (**Fig. S3B**). RT-qPCR analysis confirmed that expression of the top five DEGs such as *EIF3CL, EIF4EBP1, CARS, GARS* and* MARS* was significantly decreased with Super-EBS compared to EBS (**Fig. S3C**). To further identify early events associated with regulation of protein translation by Super-EBS, we screened phospho-protein antibody arrays that included kinases regulating protein translation. These studies used whole-cell lysates from A549 cells treated with vehicle (DMSO) or Super-EBS (4 µM for 6 h). These screens indicated that phosphorylation of the T389 residue of S6K1 reproducibly showed a decrease in intensity in protein extracts from cells treated with Super-EBS relative to vehicle-treated cells (**Fig. 3A**). To validate phospho-S6K1 (p-S6K1) inhibition by Super-EBS, we tested various lung cancer and prostate cancer cell lines treated with Super-EBS for 3 or 6 h for p-S6K1 and total protein S6K1 expression by western blot analysis. As seen in **Fig. 3B**, p-S6K1 levels but not total S6K1 total protein levels were inhibited by Super-EBS relative to vehicle in all the cell lines. As it appeared from the phospho-protein antibody array that Super-EBS may also affect phospho-Akt expression in A549 cells, we performed Western blot validation studies in A549 and LNCaP cells treated with vehicle or Super-EBS. These validation experiments with authentic antibodies did not show any effect of Super-EBS on pT308 AKT, pS473 AKT, or total AKT **(Fig. S3D)**. Our studies reemphasize the importance of validating the antibody array results using authentic antibodies in Western blot analysis.

To determine whether p-S6K1 is a potential target of Super-EBS, we opted for the computer-assisted drug discovery approach and performed molecular modeling studies on the interaction of Super-EBS with p-S6K1. Molecular docking studies using the Molecular Operating Environment (MOE) software identified p-S6K1 as a potential target of Super-EBS as well as ebastine (**Fig. 3C; S3E**). As described in Methods, we used the plausible binding modes consistent with the docking scores and proximity to the T389 phosphorylation site on S6K1 to generate binding calculations. The binding structure for S6K1-Super-EBS generated by this computational model revealed Super-EBS binding near the T389 phosphorylation site on S6K1, and this interaction was predicted to prevent S6K1 phosphorylation and activation. Interestingly, ebastine was also predicted to bind to S6K1 with binding energy similar to that for S6K1-Super-EBS interaction but the interaction of ebastine with S6K1 was not expected to directly interact with the T389 phosphorylation site (**Fig. S3B**). As seen in **Fig. 3C**, the primary amino (NH_2_) group in Super-EBS acts as a “backbone donor” to S6K1 aa Ile51 (shown by arrow) that enables Super-EBS to gain close proximity to T389 and inhibit phosphorylation. In contrast to Super-EBS, EBS has an oxygen molecule instead of the primary amino group at this position that hinders close proximity with Ile51, thereby preventing any interaction with the T389 residue of S6K1. Super-EBS and EBS exhibited similar predicted binding energy, but Super-EBS but not EBS, was predicted to bind near T389 and prevent phosphorylation of S6K1. Moreover, the extensive sensitivity data from PRISM analysis of over 900 cancer cell lines predicted S6K1 as a candidate regulator of growth inhibition by Super-EBS (**Table S2**).

To validate S6K1 as a target of Super-EBS, we generated a biotinylated-Super-EBS analog (**Fig. 3D**). Next, we performed drug-target binding studies as described in Methods to determine whether biotinylated-Super-EBS binds to S6K1. These experiments indicated that Super-EBS pulled-down S6K1 but not its closely related isoform S6K2 (**Fig. 3D**). To further test the interaction of Super-EBS and S6K1, we performed pull-down experiments using purified recombinant S6K1 and Par-4 proteins. As seen in **Fig. 3E**, biotinylated-Super-EBS binds to recombinant S6K1 but not Par-4 protein. Collectively, these studies indicated that S6K1 was a primary target of Super-EBS.

### Super-EBS shows sustained inhibition of pS6K1 levels

**Figure 4:**
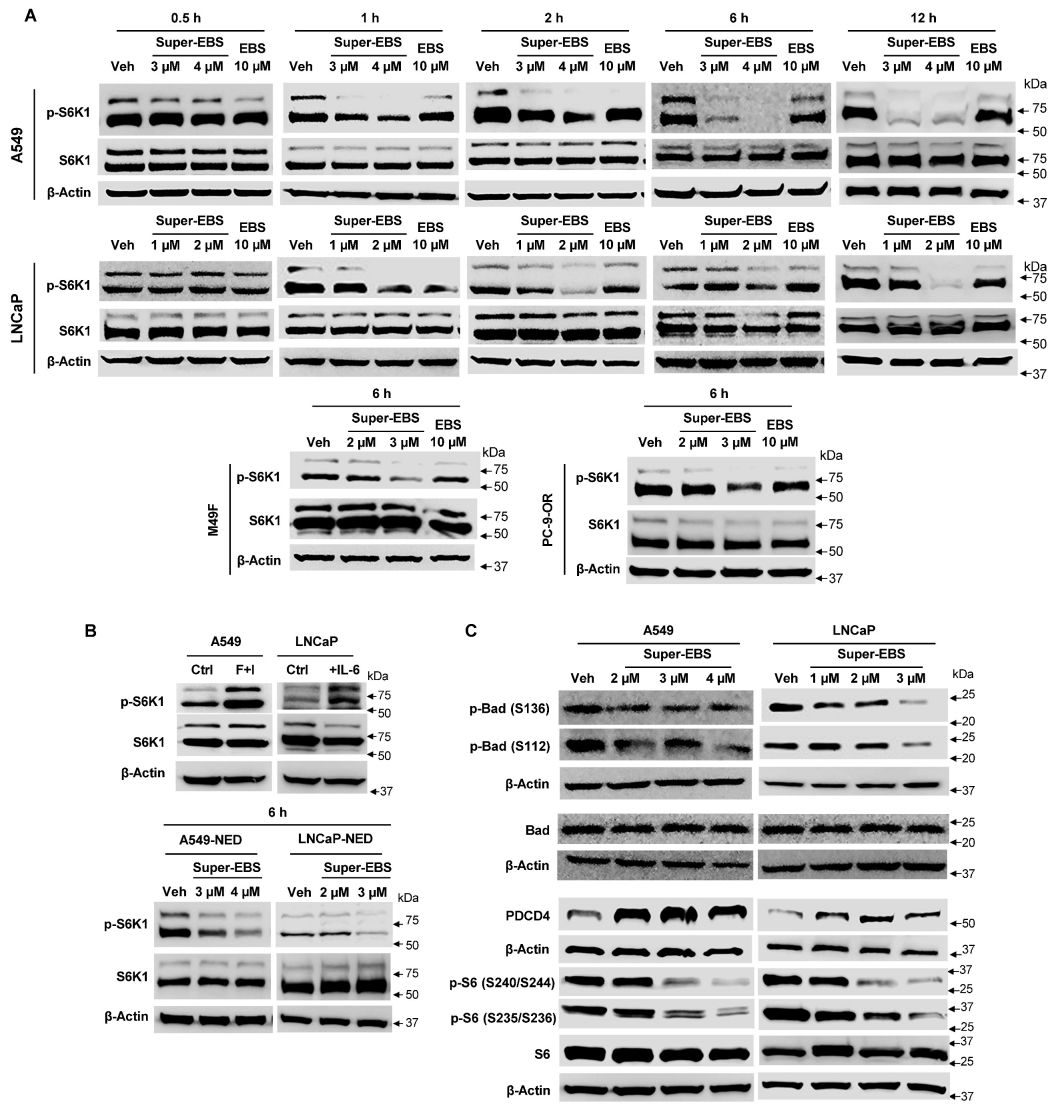
** Sustained down-regulation of p-S6K1 with Super-EBS. (A)** Super-EBS but not EBS shows sustained inhibition of pS6K levels. Cancer cell lines (A549, PC-9-OR, LNCaP, M49F) were treated with Super-EBS or EBS for various time intervals and whole-cell lysates were subjected to western blot analysis for p-S6K1 and total S6K1 protein. **(B)** Super-EBS inhibits pS6K levels in neurodifferentiated cells. A549 or LNCaP cells were induced to undergo NED by treatment with forskolin + IBMX (F+I, 0.5 mM of each for 96 h) or IL-6 (50 ng/ml for 6 days) or control vehicle (Ctrl). Whole-cell lysates were then examined by western blot analysis for p-S6K1 and total S6K1 protein (Left Panel). Neuroendocrine differentiated (NED) A549 and LNCaP cells (A549-NED and LNCaP-NED) were treated with vehicle (V) or different concentrations of Super-EBS for 6 h in serum containing medium and whole-cell lysates were then examined for p-S6K1 and S6K1 total protein levels by western blot analysis. **(C)** Super-EBS causes a dose- and time-dependent regulation of p-S6K1 and its downstream targets. A549 and LNCaP cells were treated with various concentrations of Super-EBS or vehicle as control for 6 h and whole-cell lysates were tested for the downstream targets of p-S6K1, i.e., p-Bad (S136) and (S112), p-S6 (S235/236) and (S240/S244) and PDCD4, by western blot analysis.

To determine the kinetics of p-S6K1 inhibition by Super-EBS, we tested various therapy-sensitive and therapy-resistant cancer cell lines (A549, PC-9-OR, LNCaP, M49F) for p-S6K1 expression in response to Super-EBS and EBS. Whole-cell lysates were prepared from the cancer cell lines after treatment with EBS or Super-EBS for various time intervals and subjected to western blot analysis for p-S6K1 and total S6K1. As seen in **Figs. 4A** and** S4A**, Super-EBS induced dose- and time-dependent decrease in phospho-S6K1 (p-S6K1) levels beginning as early as 3 h and lasting well beyond 12 h of treatment. By contrast, EBS caused transient suppression of p-S6K1 that was evident after 1 h of treatment (**Figs. 4A** and** S4A**). Neither Super-EBS nor EBS inhibited the expression levels of total S6K1 protein. Moreover, Super-EBS did not inhibit the expression of p-S6K1 in normal cells (**Fig. S4B**).

As Super-EBS inhibits the growth of lung and prostate cancer cells that have undergone NED, we tested p-S6K1 levels in A549 or LNCaP cells that were induced to undergo NED following treatment with Super-EBS. Interestingly, A549-NED and LNCaP-NED cells showed increased p-S6K1 expression relative to the parent A549 and LNCaP cells, respectively (**Fig. 4B**). Importantly, Super-EBS inhibited the expression of p-S6K1 but not total S6K1 in both A549-NED and LNCaP-NED cells (**Fig. 4B**).

Phosphorylation of S6K1 results in activation of the kinase that subsequently results in alteration of its downstream substrates, including pro-apoptotic proteins Bad and PDCD4, and regulator of ribosome biogenesis rpS6. We examined whether p-S6K1 inhibition resulted in a corresponding regulatory effect on its downstream substrates. A549 and LNCaP cells were treated with various concentrations of Super-EBS or vehicle and whole-cell lysates were tested for p-S6K1 and total S6K1 as well as for the downstream targets of p-S6K1, i.e., p-Bad, p-S6 and PDCD4 by western blot analysis. As seen in **Fig. 4C**, Super-EBS inhibited the expression of phospho-Bad (S136) and (S112), and p-S6 (S235/S236) and (S240/S244), and induced the expression of PDCD4 in a dose-dependent manner in both A549 and LNCaP cells.

We also tested whether Super-EBS inhibits the expression of several other cell survival kinases, including mTORC1 substrate p-4E-BP1 that is involved in protein translation, as well as p-Erk1/2, and p-Akt. As seen in **Fig. S4C**, Super-EBS did not inhibit the expression of phospho- or total levels of 4E-BP1, Erk1/2, or Akt1. In contrast to Super-EBS that directly inhibits p-S6K1, PF-4708671, a previously reported inhibitor of S6K1, did not cause a remarkable reduction in p-S6K1 levels (**Fig. S4D**). This finding on the lack of inhibition of p-S6K1 by PF-4708671 is consistent with the insignificant loss of cancer cell viability by this compound as judged by resazurin assays (**Fig. S4E**). Collectively, these findings indicate that Super-EBS preferentially inhibits the phosphorylation of S6K1 that correlates with loss of viability in diverse cancer cells.

### Delineating the link between p-S6K1 inhibition and caspase-2 activation by Super-EBS

**Figure 5:**
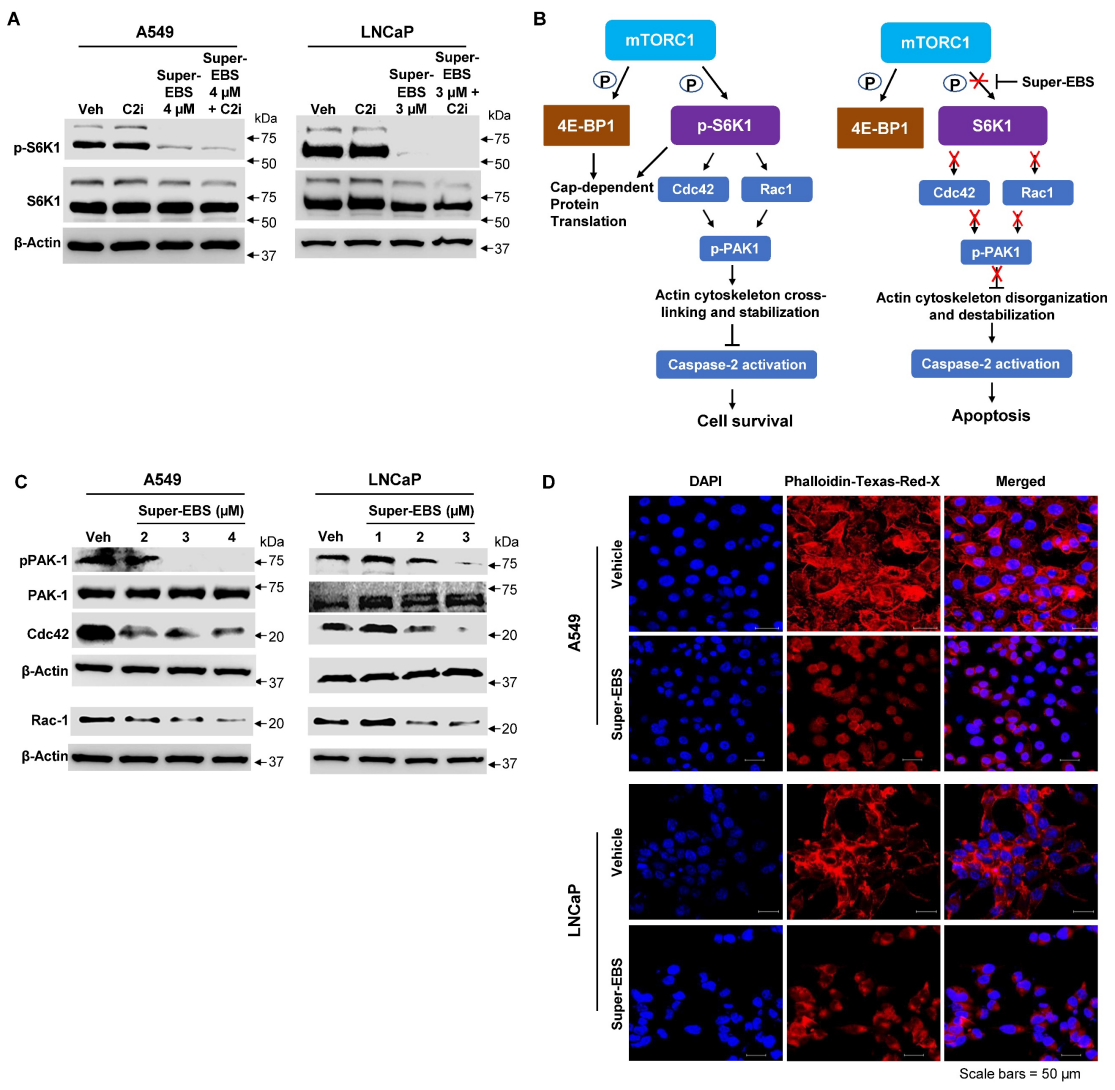
** Relationship between p-S6K1 inhibition and caspase-2 activation. (A)** Inhibition of p-S6K1 by Super-EBS is not prevented by caspase 2 inhibitor treatment. A549 and LNCaP cells were treated with vehicle or Super-EBS in the presence or absence of the caspase 2 inhibitor (C2i) (20 µM) for 6 h and whole-cell lysates were tested for p-S6K1 and total S6K1 protein levels by western blot analysis. **(B)** Link between p-S6K1 inhibition and caspase-2 activation. Schematic diagram depicting inhibition of T389 phosphorylation of S6K1 by its upstream kinase complex mTORC1 in the presence of Super-EBS, and presenting the relation between p-S6K1 and caspase 2.** (C)** Inhibition of p-S6K1 by Super-EBS causes down-regulation of Rho family GTPases and their downstream effector. A549 and LNCaP cells were treated with vehicle or the indicated concentrations of Super-EBS for 6 h and whole-cell lysates were tested for Rac-1, Cdc42 and their downstream effector p-PAK1 by western blot analysis. **(D)** Super-EBS causes actin depolymerization downstream of p-S6K1 inhibition. A549 and LNCaP cells treated with vehicle or Super-EBS (3 µM for A549 cells and 2 µM for LNCaP cells) for 9 h and immunocytochemistry was performed using Texas Red-X Phalloidin and nuclei were counter-stained with DAPI as described in the Methods. Images shown at 20X. Scale bar 50 µm.

As Super-EBS induces caspase 2 activation as well as p-S6K1 inhibition, we sought to establish the link between these two events. Since it was plausible that caspase 2 activation may cause cleavage and degradation of p-S6K1, we examined whether caspase 2 activation occurs upstream of p-S6K1 inhibition. A549 and LNCaP cells were treated with vehicle or Super-EBS in the presence or absence of caspase 2 inhibitor and whole-cell lysates were tested for p-S6K1 and total S6K1. As seen in **Fig. 5A,** p-S6K1 inhibition by Super-EBS is not prevented by caspase 2 inhibitor treatment. These findings implied that caspase 2 activation occurs downstream and not upstream of p-S6K1 inhibition by Super-EBS.

Next, we sought to reconstruct the pathway linking p-S6K1 inhibition and caspase 2 activation. Previous studies have indicated that p-S6K1 stimulates the activation of members of the Rho family of small GTPase particularly Rac-1 and Cdc42, and their downstream effector p21-activated kinase (PAK1) leading to reorganization of the actin cytoskeleton 45. Moreover, in separate studies, depolymerization of actin filaments was suggested to induce caspase 2 activation 46. We hypothesized a link between p-S6K1, and caspase 2 as presented in **Fig. 5B**. To determine if Super-EBS leads to down-regulation of the Rho family GTPases and their downstream effector, A549 and LNCaP cells were treated with vehicle or the indicated concentrations of Super-EBS and whole-cell lysates were tested for Rac-1, Cdc42 and p-PAK1 by western blot analysis. As seen in **Fig. 5C**, Super-EBS treatment inhibited Rac-1, Cdc42 and p-PAK1 expression levels in both cancer cell lines. Moreover, Super-EBS also induced actin depolymerization (**Fig. 5D**) but did not alter tubulin expression or organization in A549 and LNCaP cells (**Fig. S5A, B**). Collectively, these findings indicate that caspase 2 activation induced by Super-EBS occurs downstream of p-S6K1 inhibition and is associated with inhibition of Rac-1, Cdc42 and p-PAK1 and actin depolymerization.

### Inhibition of p-S6K1 is essential for Super-EBS mediated growth inhibition

**Figure 6:**
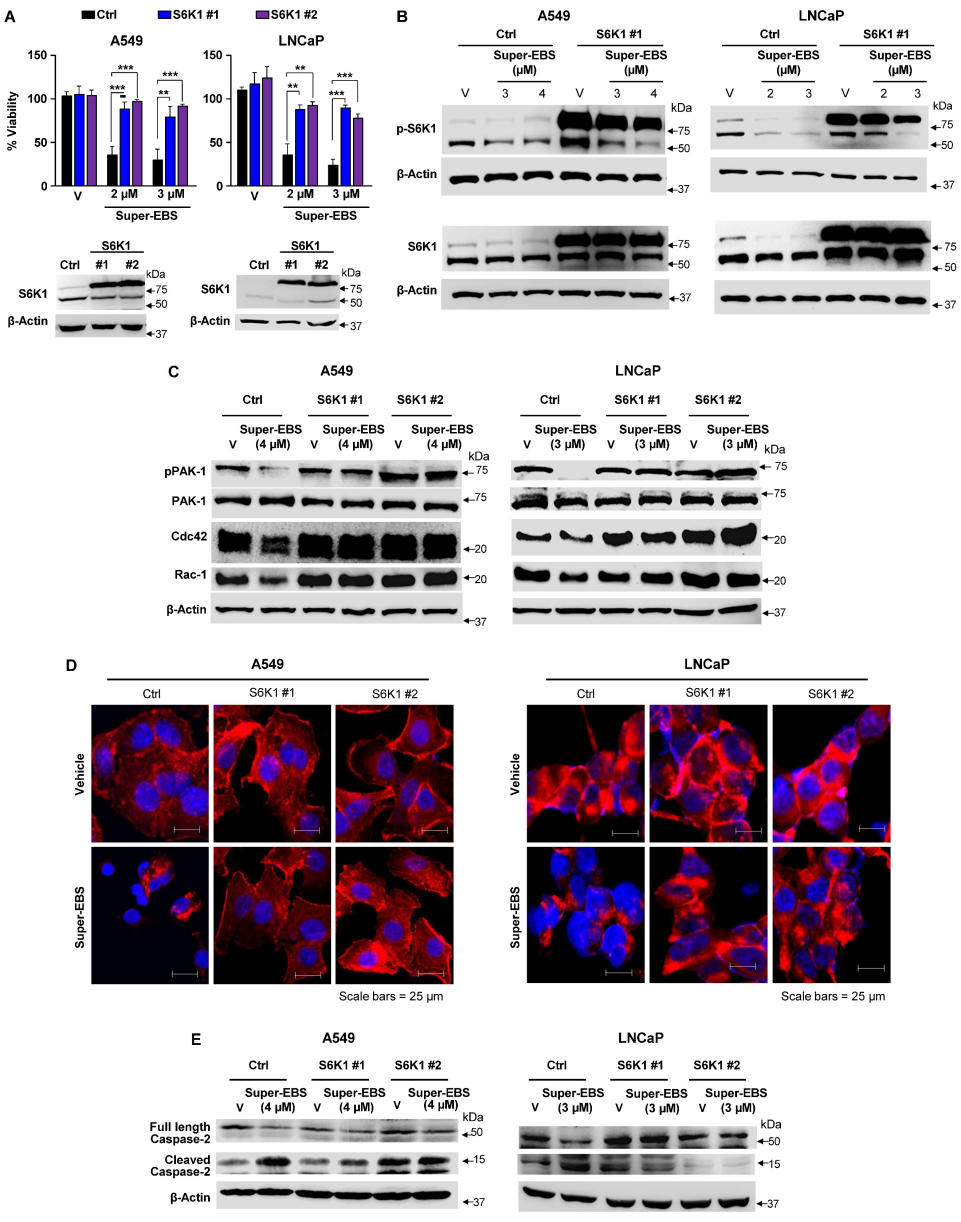
** Inhibition of p-S6K1 is essential for Super-EBS mediated cell death. (A)** S6K1 restoration reverses the effect of Super-EBS. A549 and LNCaP cell lines were transfected with S6K1 expression plasmid or control vector. The expression of ectopic S6K1 in two stably transfected clones (S6K1#1 and #2) of each cell line was verified by western blot analysis (Lower Panel). The cells were then treated with vehicle or the indicated concentrations of Super-EBS for 24 h and tested for cell viability by resazurin assays (Upper Panel). **(B)** Phosphorylation of ectopically expressed S6K1 but not total S6K1 protein levels is inhibited by Super-EBS. Transfected clones (#1) of A549 and LNCaP cells expressing ectopic S6K1 were treated with vehicle or the indicated concentrations of Super-EBS for 6 h and the effect on ectopic and endogenous p-S6K1 and total S6K1 levels was determined by western blot analysis. **(C)** Ectopically expressed S6K1 inhibits downregulation of Rac-1 and Cdc42 in response to Super-EBS. Transfectant clones (#1 and #2) of A549 and LNCaP cells expressing ectopic S6K1 were treated with vehicle or the indicated concentrations of Super-EBS for 6 h and the effect on Rac-1, Cdc42, p-PAK1 and PAK1 were determined by western blot analysis. **(D)** Ectopically expressed S6K1 inhibits disorganization of actin cytoskeleton in response to Super-EBS. Transfectant clones (#1 and #2) of A549 and LNCaP cells were treated with vehicle or Super-EBS (3 µM for A549 cells and 2 µM for LNCaP) for 9 h and immunocytochemistry was performed using Texas Red-X Phalloidin and nuclei counter-stained with DAPI. Images shown at 20X. Scale bar 25 μm. **(E)** Caspase-2 is not activated in response to Super-EBS in transfectants ectopically expressing S6K1. Transfectant clones (#1 and #2) of A549 and LNCaP cells expressing ectopic S6K1 were treated with vehicle or the indicated concentrations of Super-EBS for 12 h and the effect on caspase-2 was determined by western blot analysis.

To determine whether p-S6K1 inhibition was essential for the growth inhibitory action of Super-EBS, we generated stably transfected clones of A549 and LNCaP cell lines that over-expressed S6K1 and tested them for sensitivity to Super-EBS. As seen in **Fig. 6A**, the growth inhibitory action of Super-EBS in both the cell lines was significantly reversed by over-expression of S6K1. Interestingly, ectopically expressed p-S6K1 but not total S6K1 in the transfectants was also partially inhibited by Super-EBS, a finding that implied post-translational inhibition of p-S6K1 (**Fig. 6B; Fig. S6A**). The overall levels of ectopic p-S6K1 were much higher in the S6K1 transfectants relative to the vector transfected control cells following treatment with Super-EBS (**Fig. 6B; Fig. S6A**). These findings are consistent with the lack of any effect of Super-EBS on S6K1 RNA, as judged by RT-qPCR analysis (**Fig. S6B**).

To determine if the overall levels of the ectopic S6K1 protected against the activation of caspase-2 in response to Super-EBS, A549 and LNCaP transfectants ectopically expressing S6K1 or control vector were treated with Super-EBS and whole-cell lysates were tested for Rac-1, Cdc42 and p-PAK1 by western blot analysis. As seen in **Fig. 6C**, a decrease in Rac-1, Cdc42 and p-Pak1 in response to Super-EBS was seen only in the vector-transfected control A549 and LNCaP cells and not in the cells over-expressing S6K1. Similarly, actin depolymerization and subsequent activation of caspase 2 in response to Super-EBS occurred only in control cells (**Fig. 6D, E**).

These findings indicated that S6K1 was an essential target of Super-EBS for growth inhibition of cancer cells.

### Super-EBS inhibits tumor growth in mice

**Figure 7:**
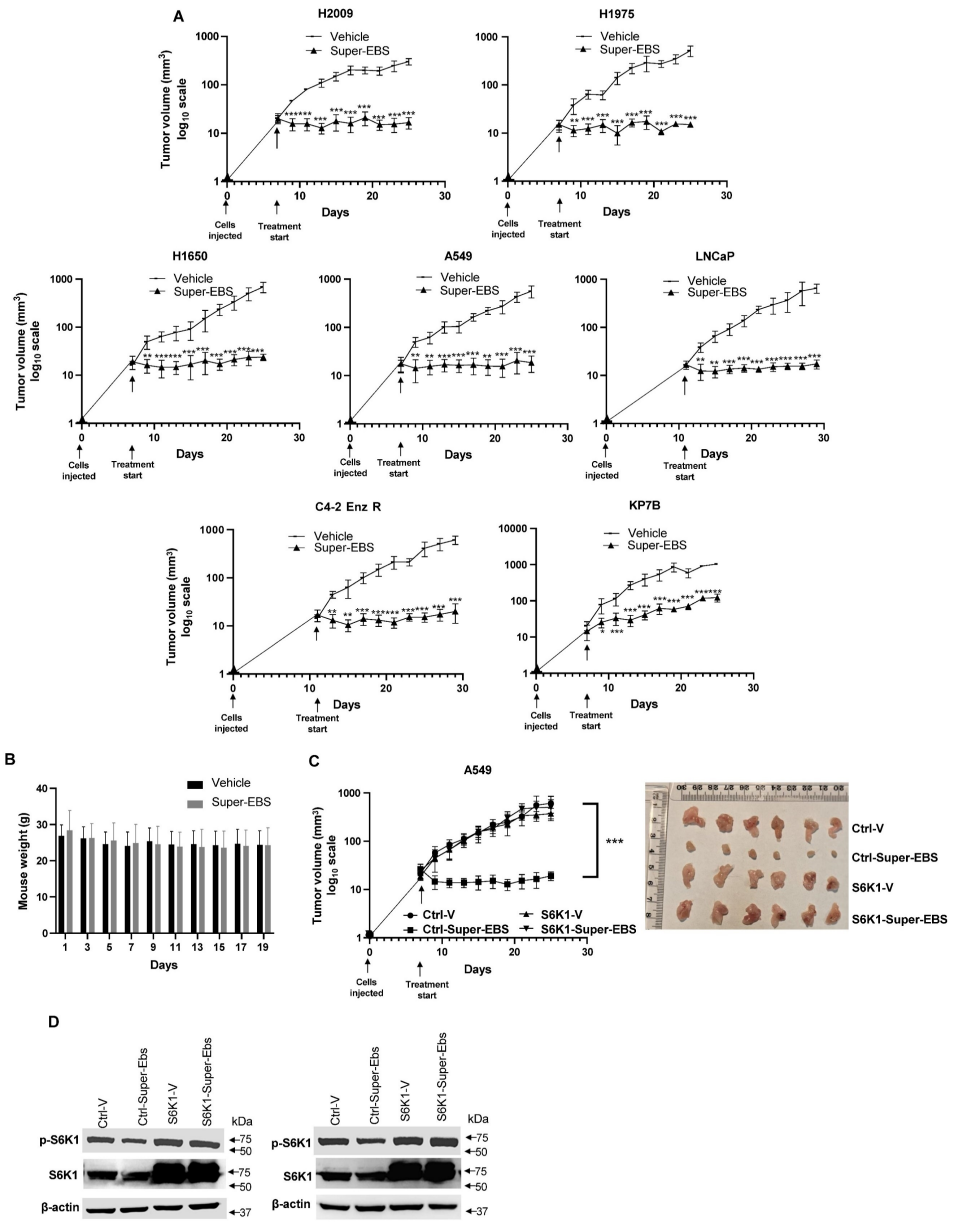
** Super-EBS inhibits tumor growth in mice but does not induce weight loss. (A)** Super-EBS inhibits tumor growth in mice. Human lung cancer- H2009, H1975, H1650, A549, and prostate cancer- LNCaP and C42R cells were injected *via* the sub-cutaneous route in NSG mice (n=7 in each group). The tumors were measured every alternate day for 20 days using calipers and tumor volumes were calculated. Mean ± SD values are shown. *P ≤ 0.05, **P ≤ 0.01, ***P ≤ 0.001, calculated using Student's *t*-test for tumor volumes at corresponding days in the vehicle control and Super-EBS treatment groups of mice. **(B)** Weights of mice not decreased by Super-EBS treatment. Average weights (Mean + SD) of the mice treated with vehicle and Super-EBS over the treatment period are shown. The differences in the vehicle and Super-EBS groups (n=7 mice per group) were not significant as calculated by the Student's *t*-test. **(C)** Xenografts of cells with ectopically expressed S6K1 were not inhibited by Super-EBS. A549 expressing ectopic S6K1 and A549 control cells expressing empty vector were injected *via* the sub-cutaneous route in NSG mice (n=6 in each group). The tumors were measured every alternate day for 20 days using calipers and tumor volumes were calculated. (Right Panel) Tumors derived from A549-ctrl (control) and A549-S6K1 (over-expressing) cells in mice treated with vehicle or Super-EBS for 20 days were collected and photographed. A549-Ctrl-V- mice injected with control cells and treated with vehicle; Ctrl-Super-EBS- mice injected with control cells and treated with Super-EBS; S6K1-V- mice injected with cells ectopically expressing S6K1 and treated with vehicle; S6K1-Super-EBS- mice injected with cells ectopically expressing S6K1 and treated with Super-EBS. (Left Panel) Mean ± SD values are shown. **P ≤ 0.01, ***P ≤ 0.001, calculated using Student's *t*-test for tumors derived from control vector or S6K1 transfected cells in mice treated with vehicle or Super-EBS. Arrow (↓) in graph represents the first day of treatment of vehicle or Super-EBS. (**D**) Super-EBS inhibits p-S6K1 in control tumors. Tumors were harvested from mice at the end of the treatment period and examined for p-S6K1 and S6K1 expression by western blot analysis. Blots are shown for two independent sets of tumors.

To determine the effect of Super-EBS on tumor growth in mice, various human lung cancer cells and prostate cancer cells were injected in NSG mice, and the resulting tumors were tested for sensitivity to Super-EBS. As seen in **Fig. 7A**, Super-EBS caused remarkable growth inhibition in tumors arising from all the cell lines tested. Super-EBS was well tolerated: it did not cause any changes in the feeding behavior, and the weights of the mice were stably maintained over the period of the experiment (**Fig. 7B**). To determine if the presence of an intact immune system altered with action of Super-EBS, C57BL/6J mice were injected with syngeneic KP7B cells and the resulting tumors were tested for sensitivity to Super-EBS. As seen in **Fig. S7A**, Super-EBS inhibited the growth of KP7B cell-derived tumors without altering the weight of the mice. Next, we tested whether tumors arising from A549 cells that over-expressed S6K1 were sensitive to Super-EBS. As seen in **Figs. 7C and 7D**, Super-EBS failed to inhibit the growth of tumors derived from S6K1 over-expressing cells, implying that S6K1 was an essential target of Super-EBS in tumors. Consistent with the above observations, A549/S6K1 KO and LLC1/S6K1 KO cells, in which S6K1 was knocked out, failed to produce robust growth of tumors compared to the control cells when injected in mice (**Fig. S7C**). This finding suggested that S6K1 was an important target that controlled the ability of the cells to form tumors in mice and that S6K1 knockout produced an effect in tumors that was similar to inhibition of S6K1 phosphorylation with Super-EBS.

## Discussion

The present study identified Super-EBS, an analog of ebastine, as an inhibitor of cancer cells including those that exhibited intrinsic resistance mechanisms or acquired resistance to therapy. Super-EBS inhibited the growth of over 95% of the diverse tumor cell lines that displayed the intra- and inter-tumoral heterogeneity in the PRISM analysis, as well as in cell culture models of lung cancer and prostate cancer plasticity. Our studies identified phospho-S6K1 as a critical target of Super-EBS that was depleted to induce cell death. Inhibition of phospho-S6K1 by Super-EBS was associated with the inhibition of Rac-1/Cdc42/p-PAK1 expression and depolymerization of actin in the caspase-2 dependent apoptotic pathway. It is important to note that Super-EBS is more potent than its parent compound, ebastine. Additionally, unlike ebastine that induces lysosomal catastrophe 47, Super-EBS causes cell death by caspase-2 dependent apoptosis. S6K1 is activated in diverse cancers, largely by upregulation of the upstream mTORC1 kinase relative to counterpart normal/benign tissues. Phosphorylated-S6K1 is an indicator for poor prognosis for cancer patients and therapy resistance 32, 34, 37, 48. Therefore, S6K1 is an important target in cancer cells. Our studies suggested that Super-EBS targeted phospho-S6K1 in tumor cells from different lineages, in cancer cell populations that represent plasticity as evidenced by neuroendocrine differentiation and/or are resistant to conventional therapy. Moreover, tumor growth inhibition by Super-EBS was dependent on depletion of endogenous phospho-S6K1 and restoration of S6K1 reversed the action of the drug. Depletion of endogenous phospho-S6K1 was necessary for growth inhibition by Super-EBS in cell culture studies, and knockout of S6K1 inhibited tumor growth in mice. Super-EBS did not remarkably inhibit pS6K1 expression or viability in normal cells in culture, and tumor bearing mice treated with Super-EBS failed to show weight loss due to nausea and lack of appetite or signs of overt toxicity. Thus, S6K1 is a critical target in tumor cells exhibiting intrinsic and acquired resistance to therapy that may be associated with tumor cell plasticity and/or tumor heterogeneity.

Intra-tumoral heterogeneity, typically characterized by divergent genotypes and phenotypes poses a major challenge in the treatment of primary and metastatic cancer. These divergent characteristics in tumors may result from plasticity of the tumor cells and manifest as intrinsic or acquired resistance to therapy. Additionally, EMT or MET or NED which are examples of tumor plasticity involve large scale and energy intensive metabolic, cytoskeletal, and genetic changes. Mathematical modeling predicts that cell proliferation occurs at the expense of cell migration and vice versa 49. Thus, the process of cellular plasticity creates a therapeutic vulnerability in the cell proliferation pathway that can be exploited to target these cells 50.

In lung cancer, oncogenic mutations in KRAS and EGFR are associated with treatment resistance. Our studies initially utilized genetically matched lung cancer cells A549 (KRAS G12S) and its taxane-resistant derivative A549TR cells, EGFR mutant PC-9 cells and its osimertinib-resistant derivative PC-9-OR cells, as well as mouse KP7B cells that carry KRAS G12D/p53 deletion and represent a clinically relevant model of lung cancer. It is important to note that ebastine was among the top ranked drugs identified in our FDA-library screen. However, Super-EBS, a structurally modified analog of ebastine was more potent than ebastine in inhibiting the growth of the drug-sensitive, as well as drug-resistant cancer cells. Our expanded panel of cancer cells sensitive to Super-EBS included lung cancer cells carrying distinct RAS mutations, such as A549 (KRAS G12S), H1299 (NRAS Q61K), H2009 (KRAS G12A), as well as EGFR mutations H1975 (EGFR L858R,T790M) and H1650 (E746 A750del), and prostate cancer cells that were androgen-responsive (LNCaP), CRPC cells derived from LNCaP cells (C4-2), their enzalutamide-resistant derivatives (M49F and C4-2R), and CRPC CWR22Rv1 cells that are intrinsically resistant to abiraterone and enzalutamide. Androgen receptor-negative prostate cancer cells PC-3 and DU145, estrogen-receptor positive breast cancer cells MCF7 and triple-negative breast cancer cells MDA MB 231 that exhibit aggressive behaviors were also sensitive to Super-EBS. Contrarily, normal lung and prostate cells were not significantly sensitive to Super-EBS. PRISM multiplexed high throughput screening of over 900 cancer cell lines representing 45 cancer subtypes indicated that a majority of the cell lines that are genotypically and phenotypically heterogeneous were sensitive to Super-EBS. Moreover, plasticity in tumors confers aggressive phenotypic outcomes and resistance to conventional treatment, yet lung and prostate cancer neuroendocrine models of plasticity showed sensitivity to Super-EBS. Collectively, we identified a novel compound that exhibits effective anti-cancer activity against a broad range of genetically and phenotypically diverse tumor cells.

The antihistamine effects of ebastine are mediated by its metabolite, carebastine in allergy patients. Carebastine, but not ebastine, and other antihistamine drugs that act through the H1 receptor failed to inhibit the growth of the cancer cells, implying that Super-EBS functions by a mechanism independent of the H1 receptor. Also, H1 receptor antibodies did not inhibit the action of Super-EBS in lung and prostate cancer cells, further supporting the H1 receptor- independent action of Super-EBS. Together, these data indicate that Super-Ebastine does not function through the H1 receptor. However, it cannot be ruled out that Super-EBS is also metabolized *in vivo* to a Super-carebastine like molecule that exerts antihistamine effects, especially in view of the more potent effect of Super-EBS noted in KP7B tumors in immuno-competent mice relative to immuno-compromised mice (**Fig. S7A**). This is also relevant as histamine receptors were reported in different human cancers such as melanoma, colon and breast cancer and anti-allergic drugs may confer protection against tumors. Histamine has been suggested to impede immunotherapy in cancer patients 51, and further *in vivo* studies may elucidate whether Super-EBS modulates immune responses and augments immunotherapy.

Ebastine has been suggested to induce cell death and tumor growth inhibition at high micro-molar doses by a mechanism that involves lysosomal catastrophe 47. Distinct mechanisms of action have been evoked for ebastine, including inhibition of the Polycomb Group Protein, Enhancer of zester homolog 2 (EZH2), and H3K27 tri-methylation, focal adhesion kinase, VCP/p97 ATPase, or JAK2/STAT3 and MEK/ERK signaling, or activation of AMPK/ULK1 signaling in various cancer cell lines 52, 53. On the other hand, our present studies indicate that Super-EBS is more potent than ebastine and induces apoptosis by a caspase 2 dependent pathway. This action of Super-EBS is linked to inhibition of phospho-S6K1, but not total S6K1. Super-EBS directly binds to S6K1 and inhibits its phosphorylation by upstream kinases. This interaction is corroborated by computer-assisted molecular modeling studies suggesting stable binding of Super-EBS to S6K1 that, unlike ebastine, is expected to block phosphorylation of S6K1 in a sustained manner. These *in silico* observations were confirmed by experimental validation with pull-down studies and immuno-blot analysis. Sustained inhibition of phospho-S6K1 by Super-EBS is linked to depletion of the Rac-1/Cdc42/p-PAK1 pathway and depolymerization of actin resulting in caspase-2 activation. The apoptosis pathway linking Super-EBS to caspase 2 is particularly noteworthy because relative to other caspases, only a few exogenous activators of caspase 2 have been identified. Although the activation of caspase 2 induces apoptotic cell death in cancer cells by caspase 3 dependent as well as independent pathways 43, 44, cell death induced by Super-EBS is prevented by the caspase 2 inhibitor but not caspase 3 inhibitor, implying that caspase 3 activation is dispensable and caspase 2 activation is essential for the growth inhibitory action of Super-EBS. It is also important to note that Super-EBS does not inhibit the phosphorylation or expression of RPS6KB2, which is a closely related structural homolog of S6K1 and downstream target of mTORC1. This selectivity in targeting S6K1 but not S6K2 may be related to the structural differences in the N- and C-terminal regions and the ATP-binding pocket regions of these kinases. Several other key proteins, including mTORC1 substrate p-4E-BP1 that is involved in regulation of downstream cap-dependent protein translation, p-Erk1/2 that promotes cell proliferation, and p-Akt that regulates cell survival are not inhibited by Super-EBS. It is important to note that phosphorylation of 4E-BP1, a downstream substrate of mTORC1 is not inhibited by Super-EBS, implying that Super-EBS inhibits phosphorylation of S6K1 by a mechanism independent of an effect on mTORC1. Collectively, these findings indicate that phosphorylation of S6K1 in diverse cancer cells is a novel target of Super-EBS.

The action of Super-EBS on survival of cancer cells in culture and xenografts was reversed by ectopically expressed S6K1. Although ectopically expressed phospho-S6K1 was partially inhibited in the transfectants by Super-EBS, the overall levels of phospho-S6K1 were much higher in the S6K1 transfectants relative to the vector transfected control cells, rendering them resistant to the action of Super-EBS. Additionally, the over-expression of S6K1 prevents the downregulation of the Rac-1/Cdc42/p-PAK1 pathway, depolymerization of actin, and subsequent caspase 2 activation in response to Super-EBS. Consistent with the almost complete growth inhibition of diverse xenografts by Super-EBS, indicating the requirement for S6K1 in tumor growth, lung cancer cells knocked out for S6K1 by CRISPR/Cas9 targeting failed to grow tumors in mice. The knockout of S6K1 likely reprograms the cells, so they grow well in cell culture, suggesting the presence of other proteins with redundant functions that compensate for the loss of S6K1 to promote monolayer growth *in vitro*. However, S6K1 function is essential for robust tumor growth *in vivo*. This observation is consistent with the fact that Super-Ebastine inhibits the growth of tumors expressing intact S6K1 by downmodulating phospho-S6K1. Collectively, our findings imply that the cancer cells are dependent on the presence of S6K1 for robust tumor growth. Moreover, in view of the possibility that monotherapy may not be sufficient to overcome cancer resistance over an extended period of time, our future studies will investigate the long-term effects of Super-EBS treatment on tumor growth inhibition and characteristics of any resistant clones. Although our present studies identified pS6K1 as the target of Super-EBS, we cannot completely rule out the possibility of a second, yet unidentified, target of Super-EBS in cancer cells. As S6K1 knockout cancer cells continue to grow in culture, but Super-EBS inhibits S6K1 and induces apoptosis in cancer cell cultures, it is very likely that Super-EBS also inhibits other cell survival proteins to cause apoptosis in cell culture. By contrast, the inhibition of S6K1 in tumors is sufficient for tumor growth inhibition by Super-EBS. Further studies aimed at developing Super-EBS or its analogs for cancer treatment will carefully consider the reasons why previously identified inhibitors of S6K1 may have failed clinical trials (**Table S5**).

In summary, this study revealed that phosphorylation of S6K1 regulates the growth and survival of diverse cancer cells by blocking the caspase 2 dependent apoptotic pathway in a manner that is independent of any effect on its upstream kinase mTORC1 and several key kinases associated with cell survival and cell proliferation. Moreover, we identified a new compound that we termed Super-EBS that inhibits the growth of tumors from diverse cancer cell lines resistant to conventional treatments by inhibition of phospho-S6K1 but not total S6K1 protein levels. Moreover, our studies identified phospho-S6K1 as a therapeutic vulnerability that can be directly targeted by Super-EBS in diverse cancer cell lines including models of cellular plasticity. Tumor plasticity is associated with, but may not necessarily be dependent on, S6K1 activity. However, by inhibition of S6K1, Super-Ebastine induces apoptosis and inhibits the growth of tumor cells that exhibit plasticity. As cellular plasticity has been suggested to create therapeutic vulnerability in the cell proliferation pathway based on the “go or grow” principle 50, future studies may determine whether S6K1 is involved in regulating the “go or grow” decisions in models of cellular plasticity and tumor heterogeneity. Further investigations on Super-EBS and its analogs, as well as its metabolites should enable context-dependent phospho-S6K1 targeting of therapy resistance in tumors.

## Supplementary Material

Supplementary methods, figures and tables.
