# Supplementary material for: S6K1 is a Targetable Vulnerability in Tumors Exhibiting Plasticity and Therapy Resistance
Source: Int J Biol Sci. 2025 Jan 1;21(2):454–72. doi: 10.7150/ijbs.96672 (PMC11705648; doi:10.7150/ijbs.96672)
Supplement: Supplementary file 1 — Supplementary methods, figures and tables. [file ijbsv21p0454s1.pdf]

Supplementary Data

S6K1 is a Targetable Vulnerability in Tumors Exhibiting Plasticity and Therapeutic Resistance

Saptadwipa Ganguly[1], Ravshan Burikhanov[2], Vitaliy M. Sviripa[3], Sally Ellingson[4,6], Jieyun Jiang[2], Christian M. Gosser[1], David Orren[1,6], Eva M. Goellner[1,6], Gautham G. Shenoy[9], Mahadev Rao[10], John D’Orazio[5,6], Christine F. Brainson[1,6], Chang-Guo Zhan[7,8], Peter H. Spielmann[3], David S. Watt[3], and Vivek M. Rangnekar[1,2, 6] *

[1] Department of Toxicology and Cancer Biology, College of Medicine, University of Kentucky, Lexington, Kentucky, USA
[2] Department of Radiation Medicine, College of Medicine, University of Kentucky, Lexington, Kentucky, USA
[3] Department of Molecular and Cellular Biochemistry and Molecular Biology, College of Medicine, University of Kentucky, Lexington, Kentucky, USA
[4] Division of Internal Medicine, College of Medicine, University of Kentucky, Lexington, Kentucky, USA
[5] Department of Pediatrics, College of Medicine, University of Kentucky, Lexington, Kentucky, USA
[6] Markey Cancer Center, University of Kentucky, Lexington, Kentucky, USA
[7] Department of Pharmaceutical Sciences, College of Pharmacy, University of Kentucky, Lexington, Kentucky, USA
[8] Molecular Modeling and Pharmaceutical Center, College of Pharmacy, University of Kentucky, Lexington, Kentucky, USA
[9] Department of Pharmaceutical Chemistry, Manipal College of Pharmaceutical Sciences, Manipal Academy of Higher Education, Manipal, India
[10] Department of Pharmacy Practice, Center for Translational Research, Manipal College of Pharmaceutical Sciences, Manipal Academy of Higher Education, Manipal, India

*** Correspondence:**
Vivek M. Rangnekar, Ph.D. University of Kentucky, 538 Healthy Kentucky Research Building, 760 Press Avenue, Lexington, Kentucky 40536. Email: vmrang01@uky.edu

Table S1. List of top ranked compounds identified from the FDA-approved drug library screen that showed cytotoxicity against lung cancer cells.

Drug	Drug Type	Cancer Activity Reported
Ebastine	Antihistamine	+
Dinaciclib (SCH727965)	CDK inhibitor	+
KPT-330	CRM1 inhibitor	+
Cytarabine	Chemotherapy	+
Mizoribine	Immunosuppressant	+
Anidulafungin (LY303366)	Antifungal	-
Cilengitide	Integrin inhibitor	+
Nelfinavir mesylate	HIV antiviral	+
Liothyronine sodium	Hormone	-
D-Cycloserine	Antibiotic	-

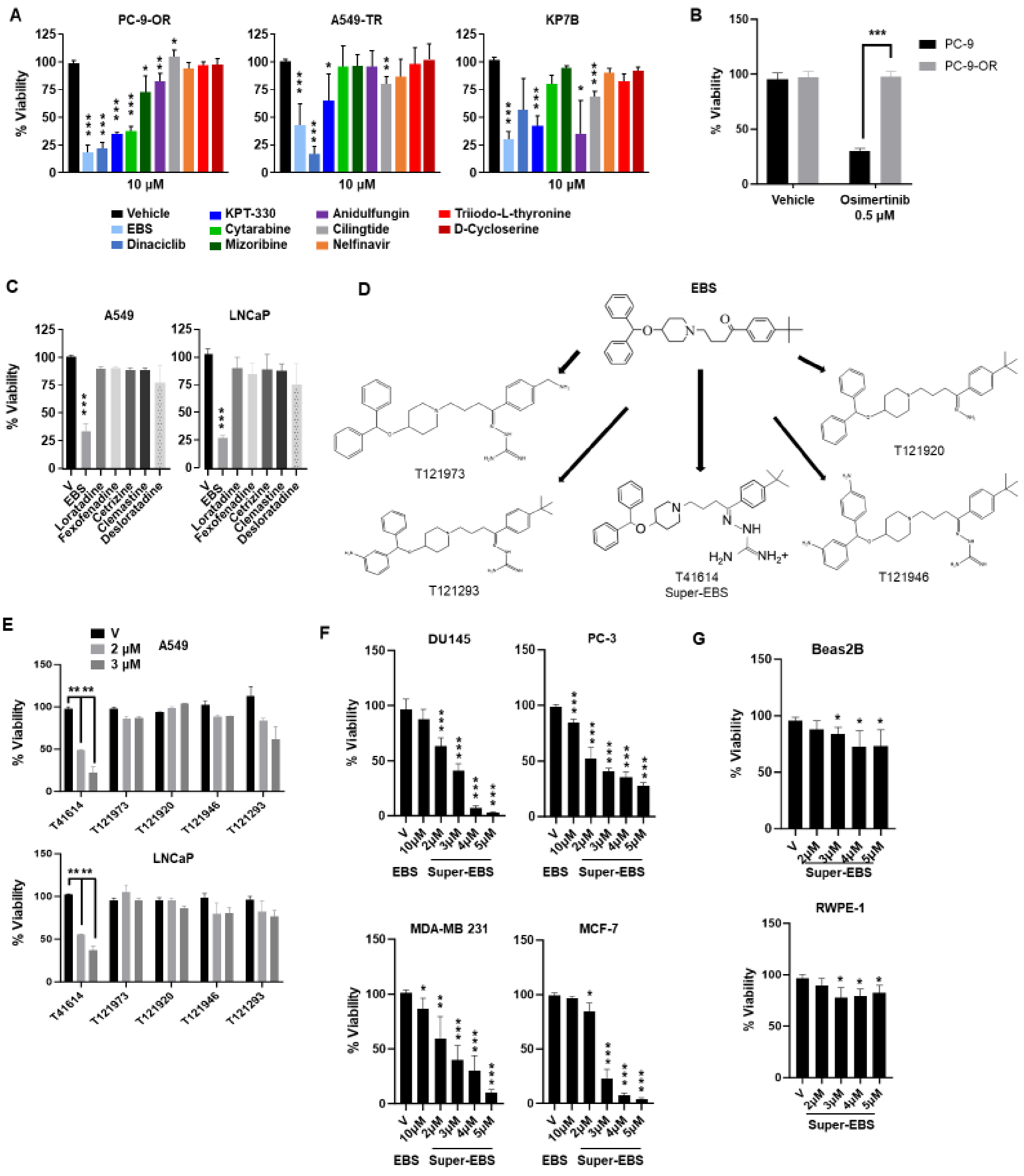
A
PC-9-OR
A549-TR
KP7B
% Viability
10 µM
Vehicle
EBS
Dinaciclib
KPT-330
Cytarabine
Mizoribine
Anidulfungin
Cilingtide
Nelfinavir
Triiodo-L-thyronine
D-Cycloserine
B
PC-9
PC-9-OR
Vehicle
Osimertinib 0.5 µM
C
A549
LNCaP
V
EBS
Loratadine
Fexofenadine
Cetrizine
Clemastine
Desloratadine
D
EBS
T121973
T121920
T121293
T41614
Super-EBS
T121946
E
V
2 µM
3 µM
A549
LNCaP
T41614
T121973
T121920
T121946
T121293
F
DU145
PC-3
MDA-MB 231
MCF-7
10µM
2µM
3µM
4µM
5µM
Super-EBS
G
Beas2B
RWPE-1

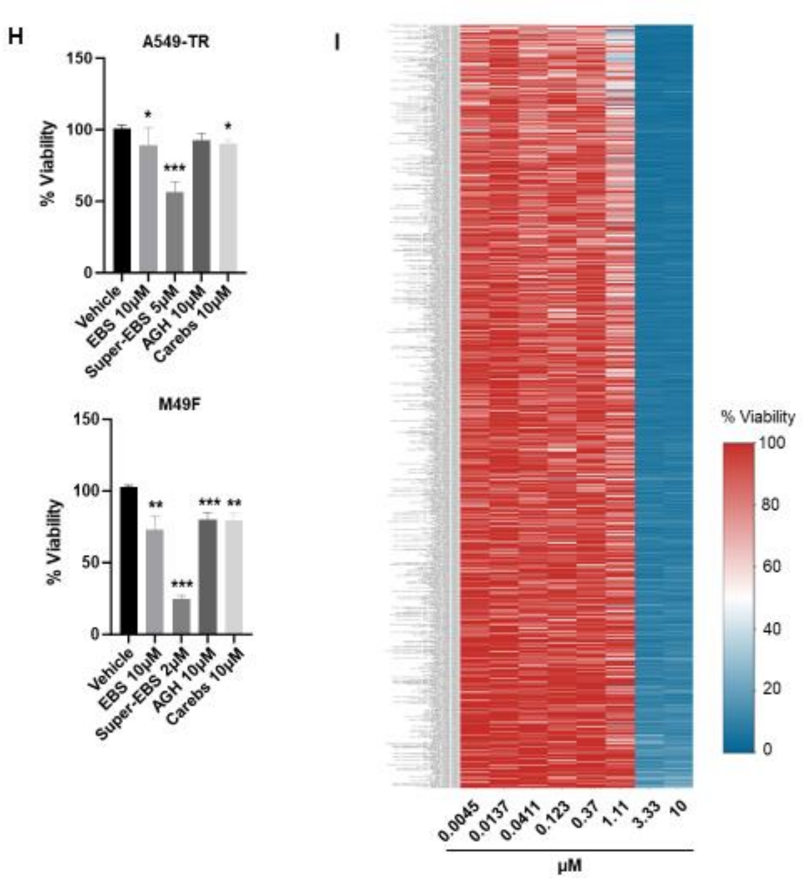

Figure S1. Identification and validation of EBS as the most effective compound in the FDA-approved drug library. (A) Ebastine showed consistent growth inhibitory effects across drug resistant lung cancer cells. Human lung cancer cells PC-9-OR (resistant to osimertinib), A549TR cells (resistant to paclitaxel), and mouse lung cancer cells KP7B (resistant to DNA damaging agents), were treated with 10 μM amounts of various drugs (listed in the **Table S1**) for 72 h and cell viability was measured by resazurin assays. Ebastine, dinaciclib and KPT-330 were identified as the top three growth inhibitory drugs and Ebastine showed consistent growth inhibitory effects across the cell lines. **(B)** Confirmation of osimertinib-resistance in the PC-9-OR cells. Osimertinib-sensitive PC-9 cells and resistant PC-9-OR lung cancer cells were treated with osimertinib for 24 h and cell viability was determined by resazurin assays. **(C)** Growth inhibitory activity of antihistamines. Lung cancer and prostate cancer cells were treated with the indicated anti-histamines in 10 μM concentrations for 72 h and cell viability was quantified by resazurin assays. **(D)** Ebastine and its analogs. Chemical structures of Ebastine and its analogs that were synthesized as described in Methods. **(E)** T41614 was the most effective analog of Ebastine in inducing growth inhibition of cancer cells. Lung cancer and prostate cancer cells were treated with different concentrations of Ebastine analog for 24 h and cell viability was quantified by resazurin assays. **(F)** Super-EBS is much more potent in growth inhibition of diverse cancer cell lines. Androgen-receptor negative prostate cancer cell lines DU145 and PC-3, triple-negative breast cancer cells MDA-MB-231, and estrogen and progesterone receptor positive MCF-7 cells were treated with various concentrations of Super-EBS for 24 h and cell viability was determined by resazurin assays. **(G)** Normal lung and prostate cancer cells show less sensitivity to Super-EBS. Normal lung Beas2B and prostate RWPE-1 cells were treated with different concentrations of Super-EBS for 24 h and cell viability was determined by resazurin assays. **(H)** Super-EBS is more effective than Ebastine in inhibition of therapy-resistant cancer cells. Paclitaxel-resistant lung cancer cells A549-TR and

enzalutamide-resistant prostate cancer cells M49F were treated with EBS (10 µM), Super-EBS (5 µM for A549-TR, 2 µM M49F), amino-guanidine (AGH) (10 µM) or carebastine (Carebs) (10 µM) for 24 h and tested for cell viability by resazurin assays. **(I)** Heatmap for sensitivity to Super-EBS based on PRISM analysis. Super-EBS was tested for growth inhibition of 900+ cancer cell lines using a high-throughput, multiplexed approach at the Broad Institute, MIT, MA, as described in Methods. Briefly, cells were treated at the indicated concentrations for 5 days and then cell viability and bioinformatics analysis were performed. Note that with treatment with 3.3 µM concentration of Super-EBS more than 95% of the cell lines showed less than 20% viability, suggesting its broad range of action in diverse tumors. **(H)** (A, B, D, F, G, H) Mean ± SD from three independent experiments are shown. *$P \leq 0.05$, **$P \leq 0.01$, ***$P \leq 0.001$ by the Student's *t*-test.

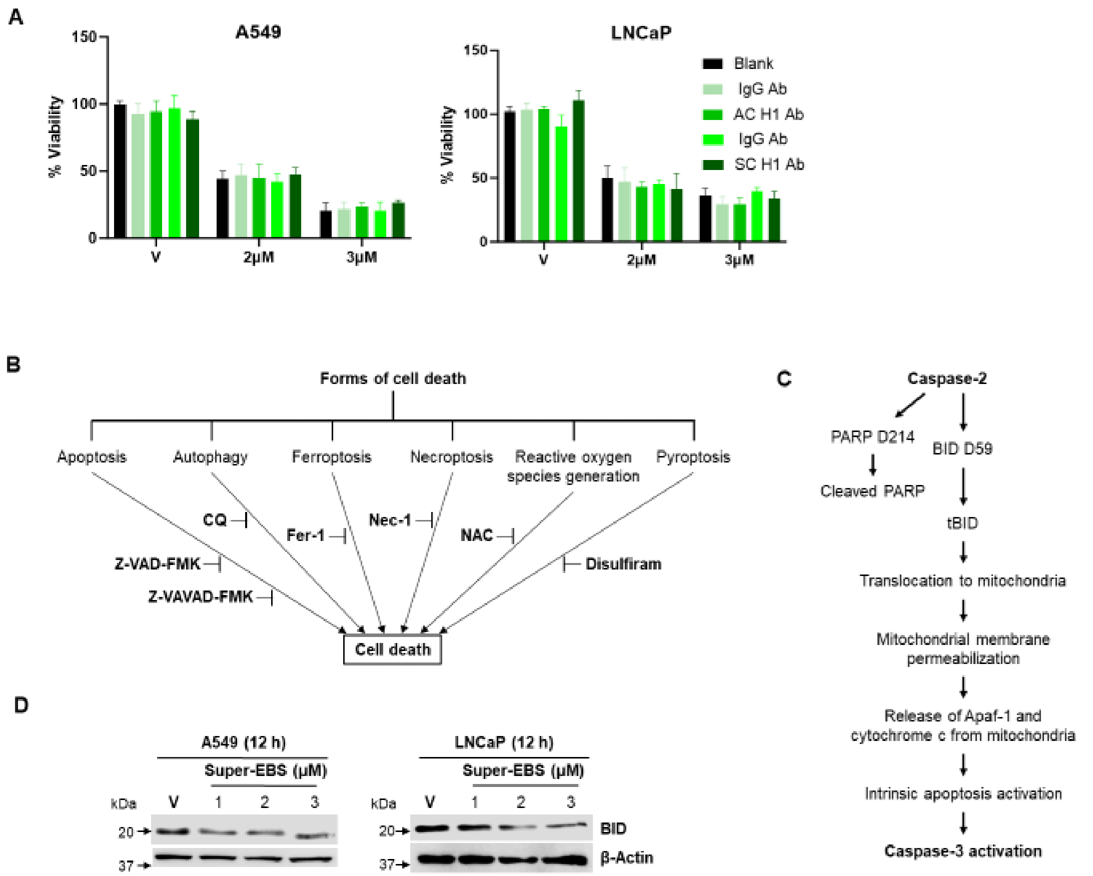

Figure S2. Super-EBS induces apoptosis. (**A**) Super-EBS does not mediate its action through H1 receptor. A549 and LNCaP cells were treated with different concentrations of Super-EBS for 24 h in the presence of isotype control and H1 receptor antibody from two different sources and viability was determined by resazurin assays. Blank- no antibody was added. IgG- mouse IgG control antibody and SC H1- monoclonal H1 receptor antibody from Santa Cruz Biotechnology, Inc, IgG- rabbit IgG control antibody and AC H1- polyclonal H1 receptor antibody from Abcam Ltd. Mean ± SD from three independent experiments are shown. *$P \leq 0.05$, **$P \leq 0.01$, ***$P \leq 0.001$ by the Student's *t*-test. **(B)** Depiction of the different cell death pathways and their corresponding pharmacological inhibitors. Cell death occurs by various pathways such as ferroptosis, necroptosis, reactive oxygen species, pyroptosis, autophagy, and apoptosis executed by different caspases. The pharmacological inhibitors for each pathway were used at previously optimized concentrations: ferroptosis [inhibitor Ferrostatin-1 (Fer-1), 2 µM], necroptosis [inhibitor Necrostatin-1 (Nec-1), 10 µM], reactive oxygen species [inhibitor N-acetyl-cysteine (NAC) 1 mM], pyroptosis [inhibitor Disulfiram, 10 µM], caspase 2 apoptosis [inhibitor Z-VDVAD-FMK, 20 µM], apoptosis by different caspases excluding caspase 2 pathway [pan-caspase inhibitor Z-VAD-FMK, 10 µM]. **(C)** Diagrammatic illustration of the apoptosis pathway downstream of caspase 2. Caspase 2 activates PARP by cleavage after amino acid D214 and BID by cleavage after amino acid D59 to generate cleaved PARP and tBID, respectively. tBID then activates caspase 3 through the mitochondrial pathway [45, 59,60]. **(D)** Super-EBS induces BID cleavage. A549 and LNCaP cells were treated with the indicated concentrations of Super-EBS for 12 h. Western blotting analysis for BID shows a decrease in total BID levels in both the cell lines.

Table S2. Genes predicted by PRISM analysis to be associated with Super-Ebastine effect in the cancer cells.

Gene	Correlation	q-value	Description
SENP1	-0.221	0.00643	Encodes a cysteine protease that specifically targets members of the small ubiquitin-like modifier (SUMO) protein family.
RPS6KB1	-0.215	0.0229	Encodes a member of the ribosomal S6 kinase family of Ser/Thr kinases. Activity of this gene has been associated with human cancer.
ZNF207	-0.203	0.0499	Kinetochore- and microtubule-binding protein that plays a key role in spindle assembly
SKP2	-0.2	0.0506	Substrate recognition component of a SCF E3 ubiquitin-protein ligase complex which mediates the ubiquitination and subsequent proteasomal degradation of target proteins
DGAT2	-0.229	0.0506	Essential acyltransferase that catalyzes the terminal and only committed step in triacylglycerol synthesis
SLC2A7	-0.22	0.0577	Probable sugar transporter
TBC1D9B	-0.189	0.0577	May act as a GTPase-activating protein for Rab family protein
HMHB1	-0.297	0.073	Precursor of the histocompatibility antigen HB-1

Genes predicted to contribute to Super-EBS sensitivity. Several genes were predicted as potential targets based on the *in-silico* analysis of the growth inhibition data from the PRISM high-throughput screen of Super-EBS. The q-value, which is an adjusted p-value taking into consideration the false discovery rate, is indicative of statistical significance.

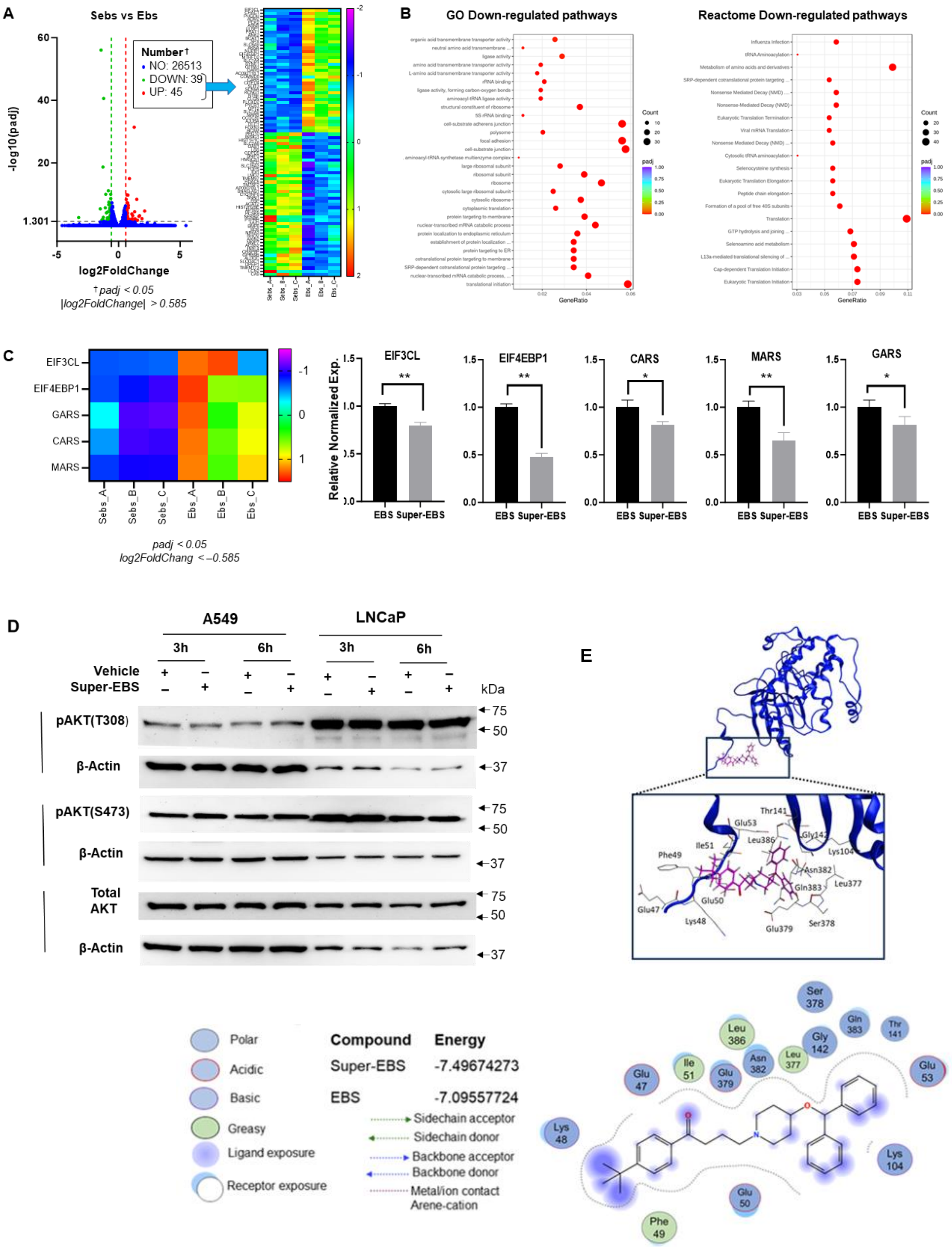

A
Sebs vs Ebs
-log10(padj)
log2FoldChange
Number†
NO: 26513
DOWN: 39
UP: 45
1.301
† padj < 0.05
|log2FoldChange| > 0.585
B
GO Down-regulated pathways
Reactome Down-regulated pathways
Count
padj
GeneRatio
C
EIF3CL
EIF4EBP1
GARS
CARS
MARS
Sebs_A
Sebs_B
Sebs_C
Ebs_A
Ebs_B
Ebs_C
padj < 0.05
log2FoldChang < –0.585
Relative Normalized Exp.
EBS
Super-EBS
D
A549
LNCaP
3h
6h
Vehicle
Super-EBS
kDa
pAKT(T308)
β-Actin
pAKT(S473)
Total AKT
75
50
37
E
Polar
Acidic
Basic
Greasy
Ligand exposure
Receptor exposure
Compound
Energy
Super-EBS
-7.49674273
EBS
-7.09557724
Sidechain acceptor
Sidechain donor
Backbone acceptor
Backbone donor
Metal/ion contact
Arene-cation

Figure S3. Differential expression analysis of RNA-Seq data. (A) Differential gene expression profile of Super-EBS versus EBS. (Left Panel) Volcano plot of differentially expressed genes (DEGs) for the Super-EBS versus EBS group. The red dots (log2FoldChange >0.585) and green dots (log2FoldChange < -0.585) represent statistically significant DEGs at FDR < 0.05 [i.e., − log10(padj) > 1.301]. (Right Panel) Heatmap of statistically significant DEGs is shown. **(B)** Pathway analysis of Super-EBS compared with EBS. Dot plots of Gene Ontology (GO) (Left Panel) and Reactome (Right Panel) enrichment pathway analysis of down-regulated DEGs are presented. The size of the dot is based on gene count enriched in the pathway, and the color of the dot shows the pathway enrichment significance. **(C)** Verification of top differentially expressed genes regulated by Super-EBS versus EBS. (Left Panel) Heatmap of top 5 statistically significant DEGs involved in down-regulation of the protein translation pathway is shown. (Right Panel) Validation of the top 5 DEGs' expression was performed by RT-qPCR analysis. $^*P \leq 0.05$, $^{**}P \leq 0.01$. **(D)** A549 and LNCaP cells were treated with vehicle or Super-Ebastine (4 μM for A549 cells and 3 μM for LNCaP cells, as in **Fig. 3B**) for the indicated time periods and whole-cell lysates were subjected to Western blot analysis for pT308 AKT, pS473 AKT, or total AKT using antibodies from Cell Signaling Technology (Danvers, Massachusetts, USA). **(E)** EBS is predicted to bind to S6K1 but not near its T389 phosphorylation site. Depiction of key predicted interactions between EBS and S6K1. The binding energy (kcal/mol) for Super-EBS or EBS is indicated.

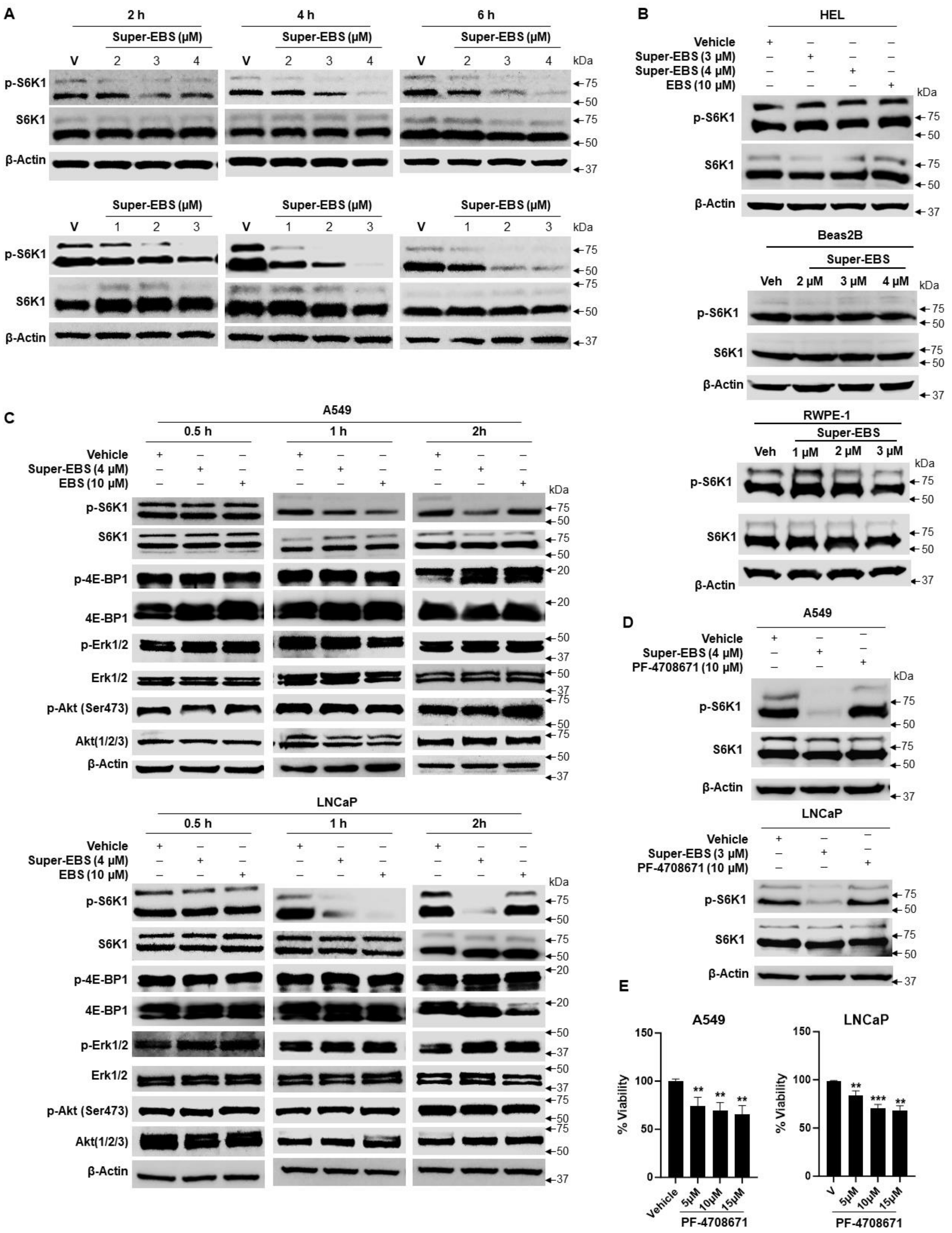
A
2 h
4 h
6 h
Super-EBS (μM)
V 2 3 4
V 1 2 3
kDa
p-S6K1
S6K1
β-Actin
75
50
37
B
HEL
Vehicle
Super-EBS (3 μM)
Super-EBS (4 μM)
EBS (10 μM)
Beas2B
Super-EBS
Veh 2 μM 3 μM 4 μM
RWPE-1
Veh 1 μM 2 μM 3 μM
C
A549
LNCaP
0.5 h
1 h
2h
Vehicle
Super-EBS (4 μM)
EBS (10 μM)
p-4E-BP1
4E-BP1
p-Erk1/2
Erk1/2
p-Akt (Ser473)
Akt(1/2/3)
20
D
Super-EBS (3 μM)
PF-4708671 (10 μM)
E
A549
LNCaP
% Viability
150
100
50
0
Vehicle
V
5μM
10μM
15μM
PF-4708671
**
***

Figure S4. Dose-dependent down-regulation of p-S6K1 by Super-EBS in cancer cells but not in normal cells. (A) Super-EBS causes a dose- and time-dependent decrease in p-S6K1. A549 and LNCaP cells were treated with various concentrations of Super-EBS or vehicle (V) as control for the indicated time intervals and whole-cell lysates were tested for p-S6K1 and total S6K1 protein levels by Western blot analysis. **(B)** Super-EBS does not inhibit p-S6K1 expression in normal cells. Normal lung fibroblasts HEL were treated with Super-EBS, EBS or vehicle, and whole-cell lysates were tested for p-S6K1 and total S6K1 by Western blot analysis. Normal epithelial lung (Beas2B) and prostate (RWPE-1) cells were treated with different concentrations of Super-EBS or vehicle (Veh) and whole-cell lysates were tested for p-S6K1 and total S6K1 by Western blot analysis. **(C)** Super-EBS inhibits the expression of p-S6K1 but not several other cell survival kinases. A549 and LNCaP cells were treated with Super-EBS or EBS for the indicated time intervals and whole-cell extracts were subjected to Western blot analysis for critical cell survival kinases, p-4E-BP1, p-Erk1/2, p-Akt and p-S6K1, as well as for each of the corresponding total proteins. **(D)** Super-EBS but not PF-4708671, specific inhibitor of S6K1 inhibits T389 phosphorylation of S6K1. A549 and LNCaP cells were treated with Super-EBS and PF-4708671 for 6 h and whole-cell lysates were tested for p-S6K1 and total S6K1 by Western blot analysis. **(E)** PF-4708671 does not significantly inhibit viability in 24 h. A549 and LNCaP cells were treated with different concentrations of PF-4708671 for 24 h and cell viability was determined by resazurin assays. Mean ± SD from three independent experiments are shown. *$P \le 0.05$, **$P \le 0.01$, ***$P \le 0.001$ by the Student's *t*-test.

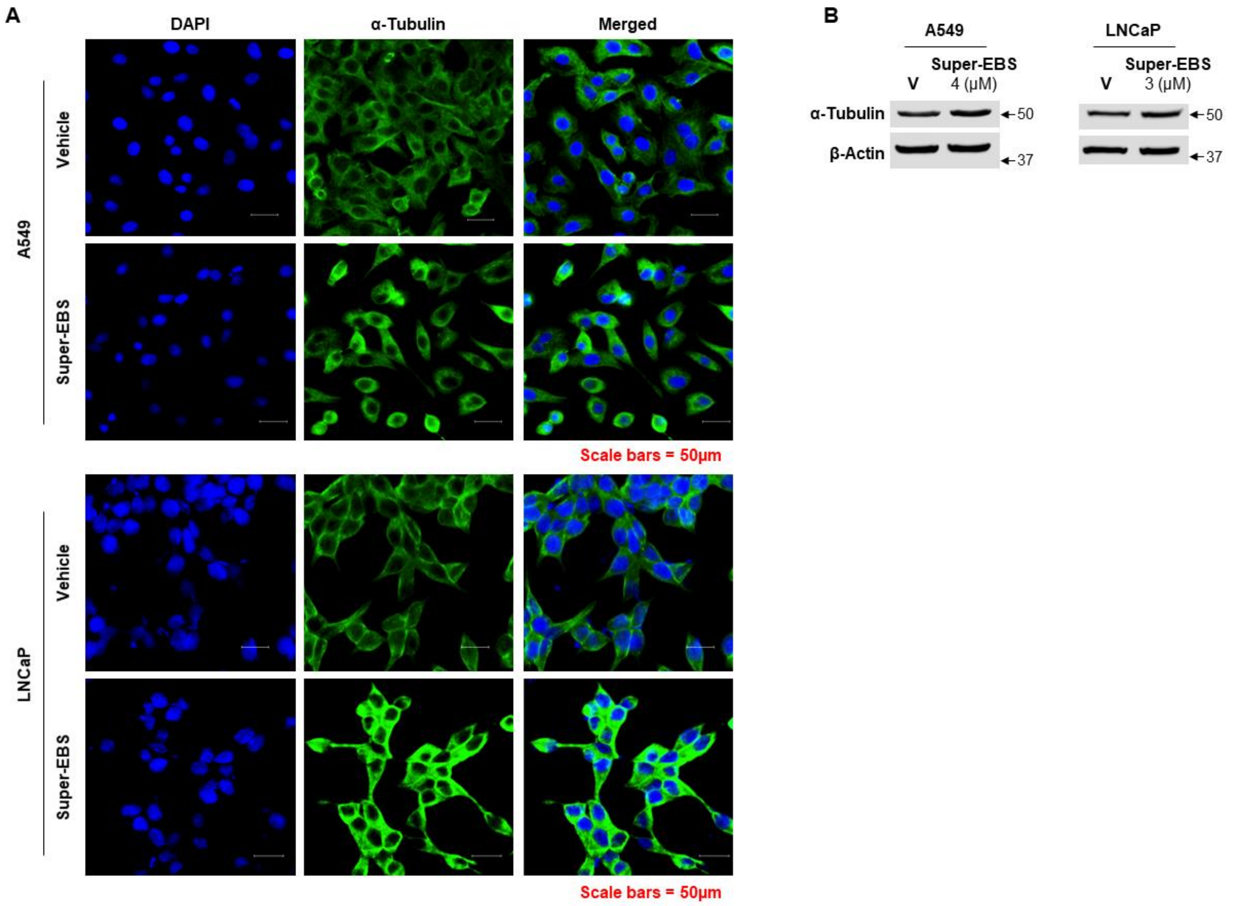

Figure S5. Super-EBS does not alter α-tubulin organization in the cells. (A) The organization of α-tubulin is not altered by Super-EBS. A549 and LNCaP cells were treated with vehicle or Super-EBS (3 µM for A549 cells and 2 µM for LNCaP cells) for 9 h and immunocytochemistry was performed using anti-α-tubulin antibody and nuclei were counter-stained with DAPI as described in the Methods. Images shown at 20X. Scale bar 50 µm. **(B)** Total α-tubulin levels are not altered by Super-EBS. A549 and LNCaP cells were treated with vehicle or Super-EBS (4 µM for A549 and 3 µM for LNCaP) for 6 h and α-tubulin levels were measured by Western blot analysis.

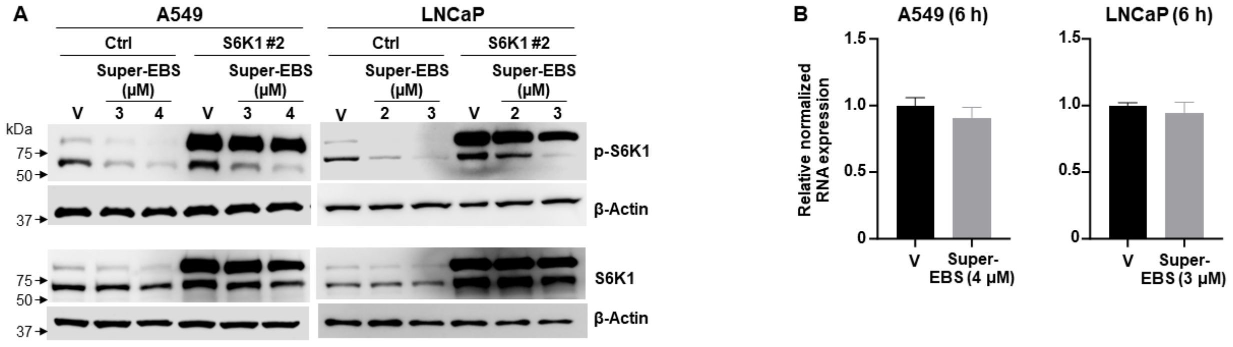

Figure S6. Super-EBS alters the phosphorylation of S6K1 without affecting the total protein. (A) Phosphorylation of ectopically expressed S6K1 but not total S6K1 is inhibited by Super-EBS. Transfected clones (#2) of A549 and LNCaP cells expressing ectopic S6K1 were treated with vehicle or the indicated concentrations of Super-EBS for 6 h and changes in ectopic and endogenous p-S6K1 and total S6K1 protein levels were determined by Western blot analysis. **(B)** Super-EBS does not alter the expression of S6K1 at the transcription level. A549 and LNCaP cells were treated with vehicle or Super-EBS for 6 h and the effect on S6K1 RNA was examined by qPCR as described in Methods. S6K1 RNA levels normalized to GAPDH RNA are shown.

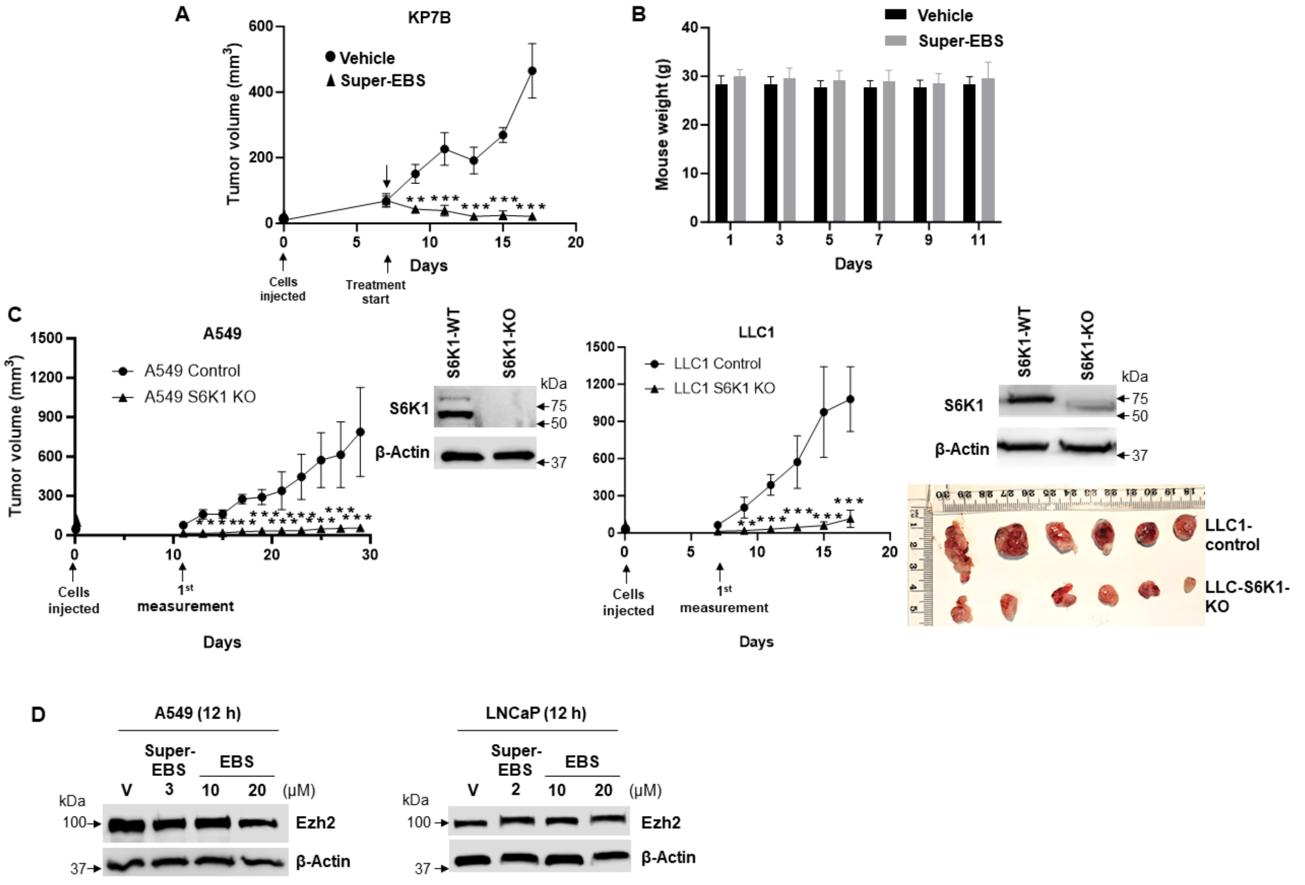

Figure S7. Super-EBS regulates tumor growth in mice but S6K1 knockout cells fail to grow tumors. (A) Super-EBS inhibits tumors in immuno-competent mice. Mouse lung cancer cells KP7B were injected *via* sub-cutaneous route in C57BL/6J (n=6). The tumors grew to 70 mm^3 in volume and then administered vehicle or Super-EBS (10 mg/kg) once daily by oral gavage. The tumors were measured every alternate day for 11 days using calipers and tumor volumes were calculated. Mean ± SD values are shown. *$P \leq 0.05$, **$P \leq 0.01$, ***$P \leq 0.001$, calculated for tumor volumes at corresponding days in the vehicle control and Super-EBS treatment groups of mice. Arrow (↓) in graph represents the first day of treatment of vehicle or Super-EBS. **(B)** Super-EBS does not cause a decrease in the weights of mice. Average weights (Mean ± SD) of the mice treated with vehicle and Super-EBS over the course of the treatment period are shown. The differences in the vehicle and Super-EBS groups (n=6 mice per group) were not significant as judged by the Student's *t*-test. **(C)** S6K1 knockout cells fail to grow tumors in mice. (Left Panel) A549 control cells and A549-S6K1 knockout (S6K1-KO) cells or LLC1 control cells and LLC1-S6K1 KO (S6K1-KO) cells were injected *via* the sub-cutaneous route in NSG mice or C57BL/6J mice respectively (n=6 in each group). Tumor measurements were made every alternate day using calipers and tumor volumes were calculated. Mean ± SD values are shown. *$P \leq 0.05$, **$P \leq 0.01$, ***$P \leq 0.001$, calculated for tumor volumes at corresponding days in the control and S6K1 KO groups of mice using Student's *t*-test. (Right Panel)

Tumors were derived from control and S6K1-KO cells, collected, and photographed. Western blot shows knock-out of S6K1 in A549 S6K1-KO and LLC-S6K1-KO cells compared to A549 control and LLC1 control cells, respectively. **(D)** EBS but not Super-EBS inhibits EZH2. A549 and LNCaP cells were treated with Super-EBS and different concentrations of EBS for 12 h. Cells were lysed and probed for EZH2 by Western blot analysis.

Supplementary Methods

Synthesis of Ebastine Derivatives

The following structural analogs of Ebastine were synthesized using the methods indicated below. Purity of the synthesized compounds was confirmed by combustion analysis or RP-HPLC, and was >90%.

Synthesis of T41614

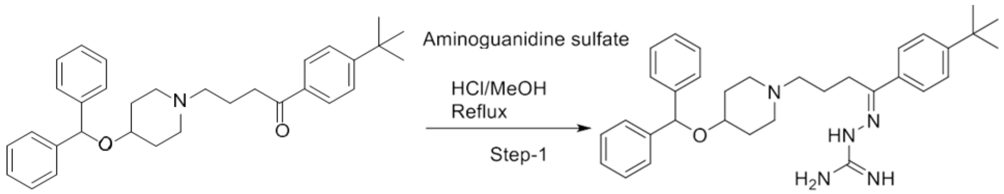

Manufacturing T41614, from Ebastine involves a straightforward condensation of Ebastine (EBS) with aminoguanidine and harkens back to a time when liquid ketones were converted to solid aryl hydrazones as part of their characterization. Ebastine from (TCI, Tokyo)

(*Z*)-2-(4-(4-(Benzhydryloxy)piperidin-1-yl)-1-(4-(*tert*-butyl)phenyl)butylidene)hydrazine-1-carboximidamide hydrochloride (T41614). To 16 mL of absolute ethanol at 80˚C in a conical centrifuge tube was added sequentially 0.66 g (5.96 mmole, 1 eq) aminoguanidine hydrochloride (Acros Organic, Carlsbad, CA), 2.8 g (5.96 mmole, 1 eq) of Ebastine (Tokyo Chemical Industry, Tokyo, Japan), an additional 6 mL of absolute ethanol, and 1.2 mL (14.2 mmole, 2.4 eq) of concentrated hydrochloric acid. After 15 min, the mixture was cooled to 25ºC and centrifuged on a desktop centrifuge at low speed for 10 min. The liquid in the centrifuge was decanted, and the remaining solid was triturated three times with 20 mL portions of absolute ethanol at 25ºC. In each of these trituration procedures, ethanol was added to the centrifuge tube containing the solid; the solid was stirred with the absolute ethanol; the mixture was centrifuged for 5 min; and the liquid was decanted. After these three trituration procedures, the resulting solid was dried under high vacuum for 12 h at 25ºC to afford 2.86 g (77%) of T41614 as a hydrochloride salt that was used in subsequent biology studies.

The free base of T41614 was prepared to obtain NMR spectrum. Twenty mg of T41614 hydrochloride was suspended in 2 mL of 0.1M aqueous sodium hydroxide solution and extracted three times with 3 mL of ethyl acetate. The combined organic phases were dried over anhydrous magnesium sulfate, filtered, and concentrated to afford T41614 free base: ^{1}H NMR (400 MHz, $CDCl_3$) δ 7.70 (m, 1H), 7.68 (m, 1H), 7.38-7.29 (m, 10H), 7.26-7.22 (m, 2H), 5.91-4.21 (br s, 4H), 5.52 (s, 1H), 3.43 (m, 1H), 2.92 (t, J = 7.8 Hz, 2H), 2.75 (bm,2H), 2.37 (t, J = 7.8 Hz, 2H), 2.11 (bm, 2H), 1.87 (bm, 2H), 1.74 (m, 4H), 1.33 (s, 9H).

Synthesis of T121293

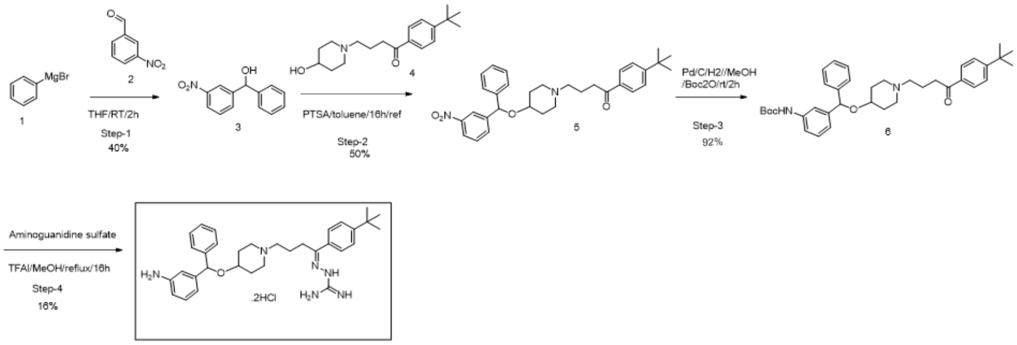

To a stirred solution of **2** (2g, 11.17 mmol) in anhydrous THF (20 ml) 2(M) phenyl magnesium bromide in ether (11.17 mL) at ice-cold condition was added and stirred at RT for 3 h. After completion of the reaction, reaction was quenched with ice-water and extracted with ethyl-acetate (100 mL x 3), dried over sodium sulphate and concentrated in vacuum. The crude was purified using flash chromatography, (eluted with 10% ethyl acetate in hexane) to give **3** (1.1 g) as a yellow liquid.

To a stirred solution of **3** (1.1 g, 4.80 mmol) in toluene (10 mL) **4** (1.45 g, 4.80 mmol) and PTSA (1.3 g, 7.20 mmol) were added at RT. Then the reaction mixture was stirred at reflux for 16 h. After completion, reaction was directly concentrated under reduced pressure to get crude compound that was purified using flash chromatography, using eluted with 5% methanol in DCM to give **5** (1.2 g) as a white solid.

To a stirred solution of **5** (1.2 g, 2.33 mmol) in MeOH (12 mL) under argon atmosphere, $(Boc)_2O$ (0.9 mL, 4.6 mmol) and 10%Pd-C (500 mg) were added to the reaction mixture and again

deoxygenated with argon for 10 min. Then the reaction vessel was back filled with hydrogen gas and the reaction mixture was allowed to stir at room temperature for 2 h under hydrogen atmosphere. After complete consumption of the starting material (monitored by TLC and LCMS), the reaction mixture was filtered through celite bed, and the filtrate was concentrated under reduced pressure to get crude compound. The crude was purified by flash chromatography with 5% methanol in DCM to afford **6** (1.2 g) as a white solid.

(Z)-2-(4-(4-((3-aminophenyl)(phenyl)methoxy)piperidin-1-yl)-1-(4-(tert-butyl)phenyl)butylidene)hydrazine-1-carboximidamide dihydrochloride (T121293)**.**To a stirred solution of **6** (200 mg, 3.42 mmol) in MeOH (2 mL) aminoguanidine sulfate (588 mg,3.42 mmol) and TFA (1 mL) were added at RT. The reaction mixture was stirred at reflux for 16 h. After completion, the reaction was concentrated under reduced pressure to get crude compound that was then purified by reverse phase prep-HPLC using HCl-buffer as an eluant to get ~35 mg (16%) of T121293 as off white solid.

^{1}H NMR (400 MHz, DMSO-d_6) δ 11.65 (s, 1H), 9.82 (br. s 1H), 7.90 (t, *J* = 7.0 Hz, 2H), 7.84 (br.s, 3H), 7.42 (d, *J* = 6.9 Hz, 2H), 7.38-7.34 (m, 4H), 7.27-7.15 (m, 2H), 7.02-6.94 (2H), 6.84 (br.s, 1H), 5.60 (s 1H), 3.69-3.55 (m, 2H), 3.46-3.42 (m, 2H), 3.36-3.32 (m, 2H), 3.27-3.23 (m, 1H), 3.15-3.10 (m, 1H), 2.97-2.91 (m, 1H), 2,32-2.19 (m, 1H), 1.98-1.78 (m, 5H), 1.30 (s, 9H). LCMS ESI (+) m/z 541.5 (M+H).

Synthesis of T121946

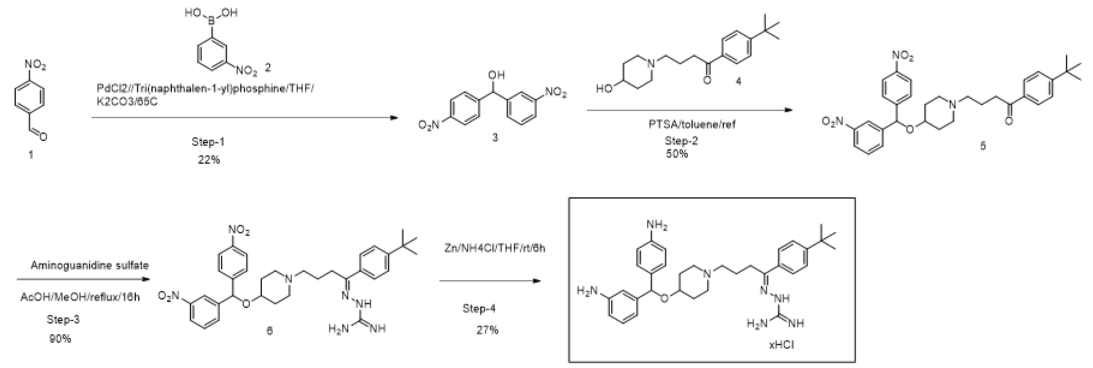

To a stirred solution of **1** (3g, 19.86 mmol) in THF (10 ml) **2** (3.3 g, 19.86 mmol) was added followed by the addition of K_2CO_3 (8.2 g, 59.58 mmol). The mixture was deoxygenated with argon and then $PdCl_2$(175 mg, 0.993 mmol) and Tri-1-naphthylphosphine (409 mg,0.993 mmol) were added under argon atmosphere at -40° C. Then the reaction mixture was heated at 65°C for 16 h. After complete consumption of the starting material (monitored by TLC and LCMS) the reaction mixture was filtered through celite bed, and the solvent was evaporated under reduced pressure to get the crude material. It was then diluted with ethyl acetate and the solution was washed successively with water and brine. Finally, the organic layer was dried over anhydrous sodium sulphate and evaporated under reduced pressure to get the crude compound, which was then purified by column chromatography over silica gel (100-200 mesh) using 30% ethyl acetate in hexane to get 1.2 g (4.37 mmol, 22%) of **6** as an off white solid.

To a stirred solution of **3** (1.2 g, 4.37 mmol) in toluene were added (10 ml) **4** (1.45 g,4.37 mmol) and PTSA (1.2 g, 6.56 mmol) at 25°C. The reaction mixture was stirred at reflux for 16 h. After complete consumption of the starting material (monitored by TLC and LCMS), the reaction mixture was concentrated under reduced pressure to get the crude compound. The crude compound was purified by flash chromatography using 5% methanol in DCM to give **5** (1.0 g) as a white solid.

To a stirred solution of **5** (500 mg, 0.893 mmol) in MeOH (5mL) aminoguanidine sulphate (154 mg, 0.893 mmol) and AcOH (1 mL) was added. The reaction mixture refluxed for 16 h with stirring. After complete consumption of the starting material (monitored by TLC and LCMS), the reaction mixture was concentrated under reduced pressure to get crude compound which was purified by triturating it with diethyl ether and pentane to get 500 mg of **6** as white solid.

(Z)-2-(4-(4-((3-aminophenyl)(4-aminophenyl)methoxy)piperidin-1-yl)-1-(4-(tert-butyl)phenyl)butylidene)hydrazine-1-carboximidamide trihydrochloride (T121946).To a stirred solution of **6** (300 mg, 0.487 mmol) in THF (2 mL) Zn (316mg,4.872 mmol) and NH_4Cl (258 mg,4.872 mmol) were added at 25°C. The reaction mixture was stirred at RT for 6 h. After complete consumption of the starting material (monitored by TLC and LCMS) the reaction mixture was filtered

through celite bed, and the solvent was evaporated under reduced pressure get the crude material. The crude compound was then purified by reverse phase prep-HPLC using HCl-buffer as an eluent to provide **90** mg (27%) of T121946 as an off-white solid.

^{1}H NMR (400 MHz, DMSO-d_6) δ 11.71 (s, 1H), 9.99 (br.s, 1H), 7.91 (t, J = 7.9 Hz, 2H), 7.86 (br.s 3H), 7.44-7.41 (m, 2H), 7.36-7.31 (m, 3H), 7.16-7.03 (m, 4H), 5.69 (s, 1H), 3.43-3.36 (m, 4H), 3.27-3.21 (m, 1H), 3,15-3.11 (m, 1H), 3.10-3.05 (m, 1H), 30.03-2.98 (m, 2H), 2.32-2.17 (m, 1H), 2.17-1.81 (m, 5H), 1.31 (s, 9H). LCMS ESI (+) m/z 556.6 (M+H).

Synthesis of T121973

To a stirred solution of **1** (2 g, 19.773 mmol) in toluene (10 ml) **2** (3.6 g,19.773 mmol) and PTSA (4.0 g, 23.728 mmol) were added at 25°C. The reaction mixture was stirred at reflux for 16 h. After complete consumption of the starting materials (monitored by TLC and LCMS), the reaction mixture was concentrated under reduced pressure to get the crude compound. Then the crude was purified by flash chromatography using 5% methanol in DCM to give **3** (2.0 g) as a white solid.

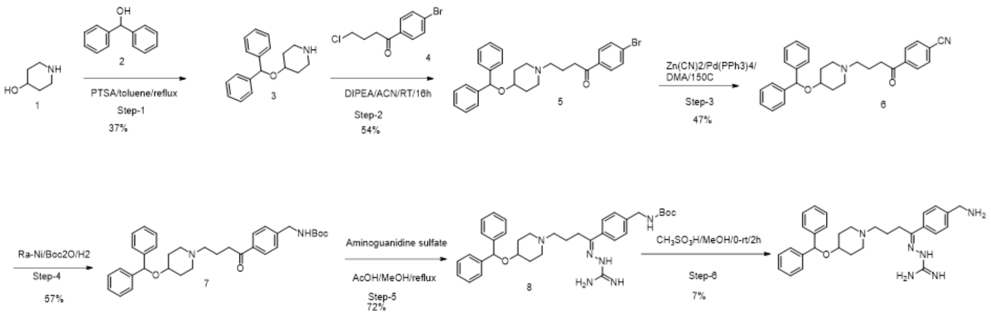

To a stirred solution of **3** (2 g, 7.48 mmol) in acetonitrile (10 ml) **4** (1.56 g, 5.98 mmol) and DIPEA (4.1 mL, 6.56 mmol) were added at 25°C. The reaction mixture was stirred at 70 ° C for 16 h. After complete consumption of the starting materials (monitored by TLC and LCMS), the reaction mixture was concentrated under reduced pressure to get the crude compound. Then the crude was purified by flash chromatography using 5% methanol in DCM to give **5** (2.0 g) as a yellow liquid.

To a stirred solution of **5** (2.0 g, 4.061 mmol) in DMA (10mL) was added $Zn(CN)_2$ (1.18 g, 10.153 mmol) followed by $Pd(PPh_3)_4$ (469 mg, 0.406 mmol). The reaction mixture was deoxygenated under argon atmosphere at 25°C. Then the reaction mixture was heated at 150°C for 16 h. After

complete consumption of the starting material (monitored by TLC and LCMS) the reaction mixture was filtered through celite bed, and the solvent was evaporated under reduced pressure get the crude material. It was then diluted with ethyl acetate, washed successively with water and brine. The organic layer was dried over anhydrous sodium sulphate and evaporated under reduced pressure to get the crude compound, which was then purified by column chromatography over silica gel (100-200 mesh) using 50% ethyl acetate in hexane to get (850 mg) of **6** as off white solid.

To a stirred deoxygenated solution of **6** (850 mg, 1.938 mmol) in methanol (10 mL) $(Boc)_2O$ (1 mL,3.876 mmol) was added followed by Raney Ni (300 mg). The reaction mixture was deoxygenated with argon for 10 min. Then, the reaction vessel was back filled with hydrogen gas and the reaction mixture was allowed to stir at room temperature for 16 h under hydrogen atmosphere. After complete consumption of the starting material (monitored by TLC and LCMS), the reaction mixture was filtered through celite bed. The filtrate was concentrated under reduced pressure to get the crude compound which was purified by flash chromatography using 5% methanol in DCM to afford **7** (600 mg) as a gummy solid.

To a stirred solution of **7** (600 mg, 1.106 mmol) in MeOH (5mL), aminoguanidine sulphate (190 mg, 1.106 mmol) and AcOH (1 mL) were added. The reaction mixture was stirred at reflux for 16 h. After complete consumption of the starting material (monitored by TLC and LCMS), the reaction mixture was concentrated under reduced pressure to get crude compound which was then triturated with diethyl ether and pentane to get 400 mg (Crude) of **8** as White solid.

(Z)-2-(1-(4-(aminomethyl)phenyl)-4-(4-(benzhydryloxy)piperidin-1-yl)butylidene)hydrazine-1-carboximidamide dihydrochloride (T121973).To a well stirred solution of **8** (400 mg, 0.667 mmol) in methanol (3 ml) methane sulfonic acid (0.04mL, 0.734 mmol) was added at 0°C under nitrogen, and the reaction mixture was allowed to stir at room temperature for 2 h under nitrogen. After complete consumption of the starting material (monitored by TLC and LCMS), the volatiles were evaporated under reduced pressure to get the crude compound. The crude compound which was then purified by

reverse phase prep-HPLC using HCl-buffer as an eluant to furnish 30 mg (7%) of T121973 as an off-white solid.

^{1}H NMR (400 MHz, DMSO-d_6) δ 11.67 (s, 1H), 10.01 (br.s, 1H), 8.34 (br.s, 2H), 8.05 (d, J = 8.2 Hz, 2H), 7.87 (br.s, 3H), 7.52 (d, J = 8.2 Hz, 2H), 7.38-7.31 (m, 8H), 7.25-7.22 (m, 2H), 5.66 (s, 1H), 4.07 (s, 2H), 3.59 (br.s, 1H), 3.34-3.18 (6H), 2.88-2.84 (m, 2H), 2.04-1.98 (m, 2H), 1.89-1.77 (m, 4H). LCMS ESI (+) m/z 499.3 (M+H).

Synthesis of T121920

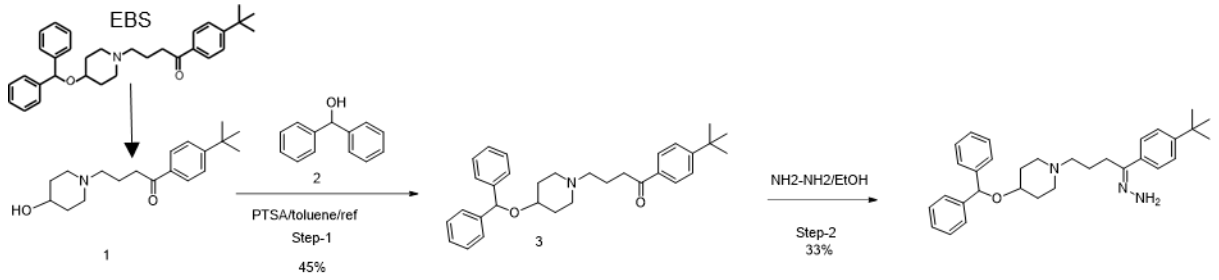

To a stirred solution of **1** (1 g, 3.296 mmol) in toluene (10 ml), **2** (1.0 g,5.932 mmol) and PTSA (850 mg, 4.943 mmol) at 25°C. The reaction mixture was stirred at reflux for 16 h. After complete consumption of the starting materials (monitored by TLC and LCMS), the reaction mixture was concentrated under reduced pressure to get the crude compound. Then the crude was purified by flash chromatography using 5% methanol in DCM to give **3** (700 mg) as a white solid.

(Z)-4-(benzhydryloxy)-1-(4-(4-(tert-butyl)phenyl)-4-hydrazineylidenebutyl)piperidine (T121920).To a stirred solution of **3** (EBS) (200 mg, 0.426 mmol) in EtOH (5 ml) hydrazine monohydrate (0.06 mL, 1.278 mmol) and acetic acid (3 µL, 0.043 mmol) were added at 25°C. The reaction mixture was stirred at reflux for 3 h. After complete consumption of the starting material (monitored by TLC and LCMS), the reaction mixture was concentrated under reduced pressure to get the crude compound. Then the crude was purified by flash chromatography using 5% methanol in DCM to give 68 mg (33%) T121920 as an off-white sticky solid.

^{1}H NMR (400 MHz, DMSO-d_6) δ 7.54 (d, J = 8.2 Hz, 2H), 7.38-7.29 (m, 10H), 7.24-7.21 (m, 2H), 6.65 (br.s, 2H), 5.63 (s, 1H), 2.7-2.62 (m, 2H), 2.57-2.50 (m, 2H), 2.20-2.15 (m, 2H), 2.02-1.96

(m, 2H), 1.88-1.81 (m, 2H), 1.59 (t, *J* = 6.6 Hz, 2H), 1.55-1.51 (m, 2H), 1.23 (s, 9H), 1.20-1.18 (m, 1H). LCMS ESI (+) m/z 484.2 (M+H).

Supplementary Materials and Methods

Chemicals, plasmids, recombinant proteins and antibodies

An FDA-approved library of 1400+ compounds (L1300) was purchased from Selleck Chemicals (Houston, TX). Chemicals such as puromycin and N-Acetyl-L-cysteine (A7250) were purchased from Sigma Aldrich (St. Louis, MO) or Fisher Scientific (Hampton, NH) unless otherwise mentioned. Ebastine was obtained from Cayman Chemical (Ann Arbor, MI) (15372-500mg) and 4, 6-diamidino-2-phenylindole (DAPI) was purchased from Vector Laboratories, Inc. (Newark, CA). The inhibitors for various cell death pathways, Ferrostatin-1 (HY-100579), Necrostatin-1 (HY-15760) and Disulfiram (HY-B0240) from MedChem Express (Malta, NY) as well as Pan Caspase Inhibitor Z-VAD-FMK (FMK001) and Caspase 2 Inhibitor Z-VDVAD-FMK (FMK003) were acquired from R&D Systems (Minneapolis, MN). The inhibitors were pre-treated for 1 h and again added after 12 h. Recombinant S6K1 protein was from Abcam Ltd. (ab167933) and Par-4 protein was from ProMab Biotechnologies.

The S6K1 plasmid (VB900005-6648jvb) was obtained from Vector Builder. A list of the antibodies used appears in the Table S3. All the antibodies were used in 1:1000 dilution except for β-Actin, which was 1:5000. The mouse (GENA931-1 mL, Sigma, St. Louis, MO) and rabbit (GENA934-1 mL, Sigma) HRP linked secondary antibody were used in 1:1000 dilution.

Table S3. List of Antibodies

Antibody	Catalog No.	Vendor
Phospho-p70 S6 Kinase (Thr389) Antibody	9205S	CST*
p70 S6 Kinase Antibody	9202S	CST
Phospho-4E-BP1 (Thr37/46) (236B4) Rabbit mAb	2855S	CST
4E-BP1 (53H11) Rabbit mAb	9644S	CST
p70 S6 Kinase 2 Antibody	14130	CST
Phospho-Akt (Ser473) Antibody	9271S	CST
Phospho-p44/42 MAPK (Erk1/2) (Thr202/Tyr204)	9101S	CST
p44/42 MAPK (Erk1/2) (137F5) Rabbit mAb	4695S	CST
Cleaved PARP (Asp214) (D64E10) XP® Rabbit mAb	5625S	CST
Phospho-Bad (Ser136) Antibody	9295S	CST

Phospho-Bad (Ser112) Antibody	9291S	CST
Cdc42 (11A11) Rabbit mAb	2466	CST
PAK1 Antibody	2602	CST
Phospho-PAK1 (Ser144)/PAK2 (Ser141) Antibody	2606	CST
Ezh2 (D2C9) XP® Rabbit mAb	5246	CST
BID Antibody	2002	CST
PDCD4 (D29C6) XP® Rabbit mAb	9535	CST
Anti-Caspase 2 Antibody, clone 10C6	MAB3501	Sigma
Monoclonal Anti-β-Actin antibody produced in mouse	A5316-100	Sigma
Anti-α-Tubulin antibody, clone DM1A	T6199	Sigma
Anti-Rac1 Antibody, clone 23A8	05-389	Sigma
Bad Antibody (C-7)	sc-8044	Santa Cruz
Akt 1/2/3 Rabbit Antibody	sc-8312	Santa Cruz
Total AKT	9272	CST
pT308 AKT	9275	CST
pS473 AKT	9271	CST
Anti-beta III Tubulin antibody - Neuronal Marker	ab18207	Abcam
Enolase 2/Neuron-specific Enolase Antibody	NB110-58870	Novus

*CST, Cell Signaling Technology

Cell viability and apoptosis assays

Cell viability was determined using resazurin assay. Cancer cells were seeded in 96-well clear bottom optical plates and after 24 h treated with the indicated concentrations of EBS or Super-EBS for 24 h. After the treatment period the media was discarded, cells were washed twice with 1X PBS (phosphate- buffered saline), media added and resazurin (R7017 Sigma-Aldrich, St. Louis, MO) assay was performed as per the manufacturer’s instructions. The absorbance was measured using a microplate reader (FLUOStar Omega, BMG LABTECH, NC) at Excitation/Emission of 544/590 nm.

Apoptotic cells were identified using Annexin V/PI kit from Invitrogen (FITC Annexin V/Dead cell apoptosis kit, V13242). In the experiment the cells were seeded in 100 mm dishes and after 24 h treated with different concentrations of Super-EBS for 24 h. Cells were harvested, and the assay was performed using manufacturer’s instructions. Measurements were done using BD Symphony A3 flow cytometer at the Markey Cancer Center Flow Cytometry and Immune Monitoring Shared Resource Facility.

Kinase antibody array assay

Culture dishes (100 mm) were seeded with A549 cells and after 24 h, they were treated with Super-EBS (4 µM) and vehicle for 6 h. Cells were harvested and lysed using the lysis buffer from the Antibody array kit (ARY003C, R&D Systems, Minneapolis, MN). Before using the lysis buffer 10 µg/mL of Aptotinin (4139, Tocris Bioscience, Bristol, UK), 10 µg/mL of Leupeptin (1167, Tocris) and 10 µg/mL of Pepstatin (1190, Tocris) were added. The protein concentration was measured using Pierce BCA protein assay kit (23227). Protein (450 µg) was used for the assay. The rest of the assay was performed as mentioned in the assay instructions and the blots were visualized using ChemiDocIT^2 Imager (UVP systems, CA).

Transfection

Cells were transfected with S6K1 expression construct or control vector (1 µg DNA) using the Plus reagent (11514015) and Lipofectamine (18324012) from Invitrogen (Waltham, MA). A549 and LNCaP cells were seeded in 6-well plates to a confluency of 60-65%. After 24 h, the cells were washed twice with 1X PBS and once with serum free media (SFM). SFM (800 µl) was added to each well. The plasmid preparations were made in SFM and added to each well. The plates were incubated overnight. After 24 h, the transfection medium was removed and 10% serum containing media was added to the cells. They were subsequently selected with 4 µg/ml and 2 µg/ml of puromycin for A549 and LNCaP cells, respectively. The positive clones were identified by performing western blot analysis for S6K1.

Generation of CRISPR/Cas knockout cells

Lung cancer A549 or LLC1 cells that were knockout for S6K1 or A549 or LLC1 cells containing the control vector were purchased from Synthego Corporation (Redwood City, CA). The cells pools were revived and cultured according to the instructions provided by the company and screened for knockout by western blot and genotyping using the primers suggested by the manufacturer. In cases where pools did not show complete knockout of the target proteins individual

Kinase antibody array assay

Culture dishes (100 mm) were seeded with A549 cells and after 24 h, they were treated with Super-EBS (4 μM) and vehicle for 6 h. Cells were harvested and lysed using the lysis buffer from the Antibody array kit (ARY003C, R&D Systems, Minneapolis, MN). Before using the lysis buffer 10 μg/mL of Aptotinin (4139, Tocris Bioscience, Bristol, UK), 10 μg/mL of Leupeptin (1167, Tocris) and 10 μg/mL of Pepstatin (1190, Tocris) were added. The protein concentration was measured using Pierce BCA protein assay kit (23227). Protein (450 μg) was used for the assay. The rest of the assay was performed as mentioned in the assay instructions and the blots were visualized using ChemiDocIT^2 Imager (UVP systems, CA).

Transfection

Cells were transfected with S6K1 expression construct or control vector (1 μg DNA) using the Plus reagent (11514015) and Lipofectamine (18324012) from Invitrogen (Waltham, MA). A549 and LNCaP cells were seeded in 6-well plates to a confluency of 60-65%. After 24 h, the cells were washed twice with 1X PBS and once with serum free media (SFM). SFM (800 μl) was added to each well. The plasmid preparations were made in SFM and added to each well. The plates were incubated overnight. After 24 h, the transfection medium was removed and 10% serum containing media was added to the cells. They were subsequently selected with 4 μg/ml and 2 μg/ml of puromycin for A549 and LNCaP cells, respectively. The positive clones were identified by performing western blot analysis for S6K1.

Generation of CRISPR/Cas knockout cells

Lung cancer A549 or LLC1 cells that were knockout for S6K1 or A549 or LLC1 cells containing the control vector were purchased from Synthego Corporation (Redwood City, CA). The cells pools were revived and cultured according to the instructions provided by the company and screened for knockout by western blot and genotyping using the primers suggested by the manufacturer. In cases where pools did not show complete knockout of the target proteins individual

clones were selected. Pools or clones were used when complete knockout out of the target protein was confirmed. At least two individual pools or clones were used in these studies.

PRISM analysis

Super-EBS at 10 mM concentration dissolved in dimethyl sulfoxide (DMSO) was tested for inhibition of cell viability at 8 different concentrations, highest being 10 μM on 900+ cancer cell lines for 5 days at the BROAD Institute (MIT/Harvard) [1]. Thereafter, the bioinformatics analysis was performed by the PRISM team to generate a potential list of genes that may contribute to Super-EBS sensitivity.

Fluorescence microscopy

To detect F-actin, the cells were treated with Super-EBS; manufacturer's instructions were followed to detect actin depolymerization using Texas Red-X Phalloidin (T7471, Invitrogen). To detect α-Tubulin (T6199, Sigma), the cells were treated with Super-EBS for the indicated time intervals and then fixed with 10% paraformaldehyde for 20 mins at room temperature. Cells were permeabilized using acetone for 15 mins at -20˚C and blocked with goat serum dissolved in 1X PBS for 1 h. The cells were incubated with primary antibody overnight at 4 °C and with secondary antibody Alexa Fluor 488 goat anti-mouse (A11001, Invitrogen, MA) for 1 h at room temperature. Nuclei were counter-stained with DAPI and imaged using a confocal microscope.

Pull down experiments

To identify protein binding to Super-EBS, pull-down experiments were performed with whole-cell lysate or recombinant proteins S6K1 or Par-4 using our previously described method [2].

RNA sequencing and enrichment analysis of differentially expressed genes

Lung cancer A549 cells were treated with EBS (10μM) or Super-EBS (Sebs, 2 μM) in triplicate for 24 h and total RNA were extracted using RNeasy mini kit (74804, Qiagen) following manufacturer's instructions. Complementary DNA (cDNA) library construction and RNA-Sequencing (RNA-Seq) were performed by Novogene (Sacramento, CA) using Illumina NovoSeq platforms with

paired-end 150 bp sequencing strategy. Bioinformatics analyses were also performed by Novogene. Briefly, RNA-Seq raw data (raw reads) were processed to obtain clean data (clean reads) by removing reads containing adapter, reads containing ploy-N and low-quality reads from raw data. The clean data were then aligned to the *Homo Sapiens* (GRCh38/hg38) reference genome using the Hisat2 [3] v2.0.5. For differential expression analysis, featureCounts [4] v1.5.0-p3 were used to count gene expression level, DESeq2 [5] v1.20.0/ EdgeR [6] v3.22.5 were used to identify differentially expressed genes (DEGs) between Super-EBS and EBS. Gene Ontology [7] (GO) enrichment analysis of differentially expressed genes was implemented by the clusterProfiler R package, in which gene length bias was corrected. GO terms with corrected P value less than 0.05 were considered significantly enriched by differential expressed genes. The Reactome database brings together the various reactions and biological pathways of human model species. Reactome pathways with corrected P value less than 0.05 were considered significantly enriched by differential expressed genes using clusterProfiler R package. The RNA sequencing results were confirmed using quantitative RT-PCR in A549 cells treated with EBS or Super-EBS for 24 h.

Real-time quantitative PCR analyses

Total RNA was extracted from the cells after treatment with vehicle or Super-EBS (4 μM for A549 and 3 μM for LNCaP) for 6 h using RNeasy mini kit (74804, Qiagen, Germantown, MD) following the manufacturer's instructions. A reverse transcription polymerase chain reaction (RT-PCR) was performed using READYSCRIPT cDNA Synthesis Mix (RDRT-100RXN, Sigma). Quantitative RT-PCR was conducted using the PERFECTA SYBR FASTMX LRX1250 (101414-286, VWR, Radnor, PA) as the detection reagent and performed on BioRad CFX96 RT-PCR system according to manufacturer's instructions. ΔΔCt methods were used to analyze the data that was normalized to the internal control, GAPDH RNA. Integrated DNA Technologies (IDT, Caralville, IA) synthesized the primers, and their sequences are indicated in **Table S4**. To confirm a single DNA duplex, a melting curve analysis of all qPCR products was conducted.

Table S4. Primer sequences.

Gene	Forward Primer (5'-3')	Reverse Primer (5'-3')
RPS6KB1 (S6K1)	GTGAACAGAGGGCCAGAAAA	TTAAGCACCTTCATGGCAAA
EIF3CL	GAGGAGGAGGACAATGAAGG	ATCAGTTCCCTTCTTGCCTC
EIF4EBP1	TCATCTATGACCGGAAATTCC	CCGCTTATCTTCTGGGCTAT
CARS	TGGAGTCAGCGCTTCAATAT	CATTGTCACAGAGGGCTTTG
MARS	ACCAAAATCACCCAGGACAT	GCATGATCGGCAGACTTTAC
GARS	TGTATGACTTTGGGCCAGTT	TGGTCAGCACGAAAACATTC
GAPDH	AAGGTCATCCCTGAGCTGAA	CCTGCTTCACCACCTTCTTG

Animal studies

NOD-Scid gamma IL2R-deleted (NSG) mice (from Jackson Laboratory, Bar Harbor, ME) were injected subcutaneously with 1.75 x 10^6 cells corresponding to the following human cancer or mouse tumor cell lines: A549, H1650, H1975, H2009, A549 S6K1-KO, A549 control, KP7B, LLC1 S6K1-KO, LLC1 control, and A549 transfected with control vector or S6K1 plasmid. We used 2×10^6 cells in experiments with LNCaP and C4-2R cell lines. Tumors were allowed to grow to a size of 25 mm^3, and the mice were split into two groups, vehicle or Super-EBS, so that the mice had similar starting tumor volumes. C57BL/6J (from Jackson Laboratory, Bar Harbor, ME) were injected subcutaneously with KP7B cells (2×10^6) dissolved in Matrigel and tumors were allowed to grow to a size of 70 mm^3. The mice were split into two groups- vehicle or Super-EBS in a manner that the mice in each group had similar starting tumor volumes.

Super-EBS (10 mg/kg) or vehicle (as a control) was administered by oral gavage. The dose and route of administration was based on outcomes from our previous mouse tumor studies with small molecules. Super-EBS was dissolved in 10% DMSO and a gavage solution containing PEG400, Vitamin E TPGS (D-alpha-Tocopherol polyethylene glycol 1000 succinate), Poloxamer 407, PBS-1X in a ratio of 25:10:1:64 was used. The solution was heated at 65 °C to generate a clear solution and given to mice daily. The tumors (and weights of the mice) were measured every other day using Vernier calipers for 20 days.

Table S5. List of direct and indirect inhibitors of S6K1 inhibitors including those that have failed clinical trials.

**Inhibitors**		**Reason for failure**
Direct inhibitors	PF-4708671	Specific S6K1 inhibitor. No clinical trial till date though it has been around for a decade [8].<br>Very high concentration (50-60 mg/kg) was required for *in vivo* tumor growth inhibition. A drastic decrease in tumor volume is not seen [9].<br>Demonstrated significant tumor reduction only in combination therapy with other drugs at high concentration (60 mg/kg or 75 mg/kg) [10] [11].<br>Leads to activation of AMPK independent of p-S6K1 inhibition by specific inhibition of mitochondrial respiratory chain Complex I [12].
	LY2584702	Specific S6K1 inhibitor. Phase 1 with dose escalation yielded no significant clinical outcomes. Also, phase 1b in combination with erlotinib and everolimus showed no evidence of tumor response [13]. Clinical trial in advanced solid tumors showed no tumor response at maximum tolerated dose [14].
	DG2, AT7867	Specific S6K1 inhibitor. Did not reach Investigational new drug (IND) status [8].
Indirect inhibitors-mTOR inhibitor	Rapalogues	Inhibition of mTORC1-S6K1 pathway leads to inhibition of negative feedback loop on IRS-1 leading to increased Akt activation and reactivation of the pathway [15].<br>In AML, mTORC2 is active therefore inhibition with rapalogues has not resulted in conclusive results.<br>Long term rapamycin treatment causes 4E-BP1 rephosphorylation possibly counteracting the effect of mTORC1 inhibition.<br>Reactivation of mTORC1 in tumor microenvironment irrespective of mTORC1 inhibition in tumor cells leads to resistance in renal cancer [16].<br>Mutation in mTOR or FKBP12 prevents optimal binding of rapalogues. Increased eIF4E, decreased 4E-BP1 expression, mutations in PP2A, and prevention of apoptosis by growth factors and cytokines are some of the mechanisms of resistance to rapalogues [17].<br>Inhibition of mTOR leads to downregulation of histone H3 and H4 acetylation leading to epigenetic changes even in tumor suppressor genes [18].

	AZD8055	mTOR inhibitor that inhibits mTORC1/2. Upregulation of ERK and p38 post AZD8055 treatment leads to modulation of 4E-BP1 mRNA pool, thus potentially hindering inhibition of protein translation [19].<br>It also induces autophagy by downregulating p62 and inhibiting ULK1 phosphorylation at Ser^{757} [20].
	SNS-032	Dual mTORC1/2 inhibitor. ABCB1 overexpression in neuroblastoma leads to resistance [21]. AML patients (14.9%) and Kasumi-1 cells were unresponsive to it [22].
Dual Akt and S6K1 inhibitor	LY2780301	Dual S6K1 and Akt inhibitor. Phase 1 clinical trial demonstrated minimal antitumor activity and S6K1 expression in epidermis did not correlate with plasma concentrations
	XL418	Dual S6K1 and Akt inhibitor. Low drug exposure led to phase 1 trial suspension [23].
	M2698	Dual S6K1 and Akt inhibitor. The progression free survival in breast cancer patients with mono or combination therapy with and without confounding mutations was only 1.4 or 2.8 months respectively [24].<br>Patients exhibited psychosis as side effects in phase I clinical trials. Potentially due to Akt inhibition in central nervous system [25].
	S34	Modified analog of M2698. Recommended for use in Covid-19. Predicted through *in silico* assays to inhibit S6K1 but further validation studies were not performed [26].

Supplementary References

1. Corsello SM, Nagari RT, Spangler RD, Rossen J, Kocak M, Bryan JG, et al. Discovering the anticancer potential of non-oncology drugs by systematic viability profiling. Nature Cancer. 2020; 1: 235-48.
2. Burikhanov R, Sviripa VM, Hebbar N, Zhang W, Layton WJ, Hamza A, et al. Arylquins target vimentin to trigger Par-4 secretion for tumor cell apoptosis. Nat Chem Biol. 2014; 10: 924-6.
3. Mortazavi A, Williams BA, McCue K, Schaeffer L, Wold B. Mapping and quantifying mammalian transcriptomes by RNA-Seq. Nature Methods. 2008; 5: 621-8.
4. Liao Y, Smyth GK, Shi W. featureCounts: an efficient general purpose program for assigning sequence reads to genomic features. Bioinformatics. 2014; 30: 923-30.
5. Love MI, Huber W, Anders S. Moderated estimation of fold change and dispersion for RNA-seq data with DESeq2. Genome Biology. 2014; 15: 550.
6. Robinson MD, McCarthy DJ, Smyth GK. edgeR: a Bioconductor package for differential expression analysis of digital gene expression data. Bioinformatics. 2010; 26: 139-40.
7. Young MD, Wakefield MJ, Smyth GK, Oshlack A. Gene ontology analysis for RNA-seq: accounting for selection bias. Genome Biology. 2010; 11: R14.
8. Artemenko M, Zhong SSW, To SKY, Wong AST. p70 S6 kinase as a therapeutic target in cancers: More than just an mTOR effector. Cancer Letters. 2022; 535: 215593.
9. Qiu Z-X, Sun R-F, Mo X-M, Li W-M. The p70S6K Specific Inhibitor PF-4708671 Impedes Non-Small Cell Lung Cancer Growth. PLOS ONE. 2016; 11: e0147185.
10. Zhang Y, Wang Q, Chen L, Yang H-S. Inhibition of p70S6K1 activation by Pdcd4 overcomes the resistance to an IGF-1R/IR inhibitor in colon carcinoma cells. Molecular Cancer Therapeutics. 2015; 14: 799-809.
11. Shen H, Wang GC, Li X, Ge X, Wang M, Shi ZM, et al. S6K1 blockade overcomes acquired resistance to EGFR-TKIs in non-small cell lung cancer. Oncogene. 2020; 39: 7181-95.

12. Vainer GW, Saada A, Kania-Almog J, Amartely A, Bar-Tana J, Hertz R. PF-4708671 activates AMPK independently of p70S6K1 inhibition. PLOS ONE. 2014; 9: e107364.
13. Hollebecque A, Houédé N, Cohen EEW, Massard C, Italiano A, Westwood P, et al. A phase Ib trial of LY2584702 tosylate, a p70 S6 inhibitor, in combination with erlotinib or everolimus in patients with solid tumours. European Journal of Cancer. 2014; 50: 876-84.
14. Tolcher A, Goldman J, Patnaik A, Papadopoulos KP, Westwood P, Kelly CS, et al. A phase I trial of LY2584702 tosylate, a p70 S6 kinase inhibitor, in patients with advanced solid tumours. European Journal of Cancer. 2014; 50: 867-75.
15. Ghosh J, Kapur R. Role of mTORC1-S6K1 signaling pathway in regulation of hematopoietic stem cell and acute myeloid leukemia. Exp Hematol. 2017; 50: 13-21.
16. Yang J, Butti R, Cohn S, Toffessi-Tcheuyap V, Mal A, Nguyen M, et al. Unconventional mechanism of action and resistance to rapalogs in renal cancer. Proceedings of the National Academy of Sciences. 2024; 121: e2310793121.
17. Kurmasheva RT, Huang S, Houghton PJ. Predicted mechanisms of resistance to mTOR inhibitors. British Journal of Cancer. 2006; 95: 955-60.
18. Makarević J, Tawanaie N, Juengel E, Reiter M, Mani J, Tsaur I, et al. Cross-communication between histone H3 and H4 acetylation and Akt-mTOR signalling in prostate cancer cells. J Cell Mol Med. 2014; 18: 1460-6.
19. Jee HY, Lee YG, Lee S, Elvira R, Seo HE, Lee JY, et al. Activation of ERK and p38 reduces AZD8055-mediated inhibition of protein synthesis in hepatocellular carcinoma HepG2 cell line. Int J Mol Sci. 2021; 22.
20. Huang S, Yang ZJ, Yu C, Sinicrope FA. Inhibition of mTOR kinase by AZD8055 can antagonize chemotherapy-induced cell death through autophagy induction and down-regulation of p62/sequestosome 1. J Biol Chem. 2011; 286: 40002-12.
21. Löschmann N, Michaelis M, Rothweiler F, Voges Y, Balónová B, Blight BA, et al. ABCB1 as predominant resistance mechanism in cells with acquired SNS-032 resistance. Oncotarget. 2016; 7.
22. Meng H, Jin Y, Liu H, You L, Yang C, Yang X, et al. SNS-032 inhibits mTORC1/mTORC2 activity in acute myeloid leukemia cells and has synergistic activity with perifosine against Akt. Journal of Hematology & Oncology. 2013; 6: 18.
23. Bussenius J, Anand NK, Blazey CM, Bowles OJ, Bannen LC, Chan DSM, et al. Design and evaluation of a series of pyrazolopyrimidines as p70S6K inhibitors. Bioorganic & Medicinal Chemistry Letters. 2012; 22: 2283-6.
24. Tsimberidou A-M, Shaw JV, Juric D, Verschraegen C, Weise AM, Sarantopoulos J, et al. Phase 1 study of M2698, a p70S6K/AKT dual inhibitor, in patients with advanced cancer. Journal of Hematology & Oncology. 2021; 14: 127.
25. Tsimberidou A-M, Skliris A, Valentine A, Shaw J, Hering U, Vo HH, et al. AKT inhibition in the central nervous system induces signaling defects resulting in psychiatric symptomatology. Cell & Bioscience. 2022; 12: 56.
26. Shechter S, Pal RK, Trovato F, Rozen O, Gage MJ, Avni D. p70S6K as a potential Anti-COVID-19 target: Insights from wet bench and in silico studies. Cells. 2024; 13: 1760.
